# Comprehensive Analysis of MEN1 Mutations and Their Role in Cancer

**DOI:** 10.3390/cancers12092616

**Published:** 2020-09-14

**Authors:** Devi D. Nelakurti, Amrit L. Pappula, Swetha Rajasekaran, Wayne O. Miles, Ruben C. Petreaca

**Affiliations:** 1 Biomedical Science Undergraduate Program, The Ohio State University Medical School, Columbus, OH 43210, USA; nelakurti.1@buckeyemail.osu.edu; 2 Computer Science and Engineering Undergraduate Program, The Ohio State University, Columbus, OH 43210, USA; pappula.1@buckeyemail.osu.edu; 3 Department of Molecular Genetics, The Ohio State University, Columbus, OH 43210, USA; rajasekaran.11@buckeyemail.osu.edu; 4 Department of Cancer Biology and Genetics, The Ohio State University Medical School, Columbus, OH 43210, USA; Wayne.Miles@osumc.edu; 5 Department of Molecular Genetics, The Ohio State University, Marion, OH 43302, USA

**Keywords:** pancreatic cancer, parathyroid cancer, mutational signatures

## Abstract

**Simple Summary:**

Cancers are characterized by accumulation of genetic mutations in key cell cycle regulators that alter or disable the function of these genes. Such mutations can be inherited or arise spontaneously during the life of the individual. The MEN1 gene prevents uncontrolled cell division and it is considered a tumor suppressor. Inherited MEN1 mutations are associated with certain parathyroid and pancreatic syndromes while spontaneous mutations have been detected in cancer cells. We investigated whether inherited mutations appear in cancer cells which would suggest that patients with parathyroid and pancreatic syndromes have a predisposition to develop cancer. We find a weak correlation between the spectrum of inherited mutations and those appearing spontaneously. Thus, inherited MEN1 mutations may not be a good predictor of tumorigenesis.

**Abstract:**

MENIN is a scaffold protein encoded by the MEN1 gene that functions in multiple biological processes, including cell proliferation, migration, gene expression, and DNA damage repair. MEN1 is a tumor suppressor gene, and mutations that disrupts MEN1 function are common to many tumor types. Mutations within MEN1 may also be inherited (germline). Many of these inherited mutations are associated with a number of pathogenic syndromes of the parathyroid and pancreas, and some also predispose patients to hyperplasia. In this study, we cataloged the reported germline mutations from the ClinVar database and compared them with the somatic mutations detected in cancers from the Catalogue of Somatic Mutations in Cancer (COSMIC) database. We then used statistical software to determine the probability of mutations being pathogenic or driver. Our data show that many confirmed germline mutations do not appear in tumor samples. Thus, most mutations that disable MEN1 function in tumors are somatic in nature. Furthermore, of the germline mutations that do appear in tumors, only a fraction has the potential to be pathogenic or driver mutations.

## 1. Introduction

**Figure 1:**
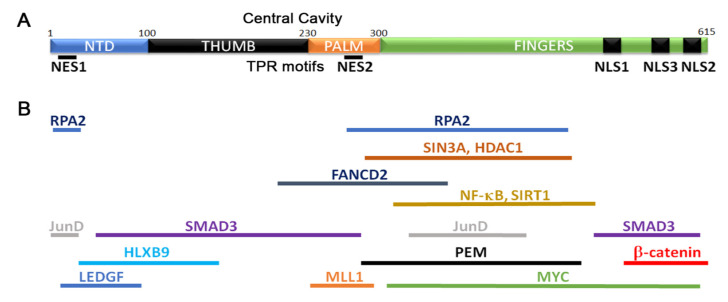
Structure and function of MENIN. (**A**) Schematic of MENIN highlighting identified domains and functional regions. (**B**) Relative positions of MENIN interaction proteins mapped on the structure in (**A**).

The tumor suppressor gene MEN1 encodes MENIN, a scaffold protein with diverse functions in cell cycle regulation, DNA repair, and gene expression [1,2]. The crystal structure of MENIN shows that it contains four major domains: an N-terminal domain (NTD), two middle thumb and palm domains, and a C-terminus Fingers domain [3,4] (Figure 1A). A central cavity in the thumb and palm domains contains several TPR (tetracopeptide repeat) motifs. Such motifs are involved in intra- and inter-molecular interactions [5] and have been proposed to facilitate MENIN interaction with various proteins. Several other structural motifs have also been identified and are discussed elsewhere [3,4]. Three nuclear localization sequences (NLS1, NLS2, NLS3) [6,7,8] and two nuclear exit sequences (NES1, NES2) have been identified [9] which show that MENIN shuttles in and out of the nucleus. MENIN interacts with a plethora of proteins [10,11] (Figure 1B). These MENIN interacting proteins function in gene expression regulation, cell cycle progression, DNA damage repair, and a variety of other processes [3,4,9,12,13,14,15,16,17,18,19,20,21,22,23,24,25,26,27,28] (Table 1). Several other interactions have been reviewed elsewhere [2]. Mapping these MENIN interacting proteins to the MENIN sequence shows that most bind the palm and fingers domains (Figure 1B).

Mutations in this gene were first identified in the multiple endocrine neoplasia type 1 syndrome [29,30]. Subsequent analysis revealed that MEN1 alterations are also found in a subset of pancreatic endocrine tumors [31,32,33] and pituitary adenomas [34], as well as several other cancers [35,36]. In addition to its independent function, MEN1 also interacts with the lysine methyltransferase KMT2A (previously MLL1), and this interaction contributes to the oncogenic properties of KMT2A [37,38,39]. KMT2A is a proto-oncogene frequently activated by chromosomal re-arrangements and acts as genetic driver in a number of leukemias [40].

MEN1 mutations can be inherited (germline or hereditary) or be somatic [41]. Inherited mutations are autosomal dominant [42] and, rather than focusing on key residues, appear equally distributed along the coding sequence [29,41,42,43,44,45,46]. Similarly, analysis of over 17,000 somatic mutations within MEN1 found that the vast majority of these mutations were distributed more or less evenly throughout the entire coding region [1]. This analysis did, however, identify 9 amino acid positions with a higher mutation frequency (hotspots), suggesting that a bias for key residues may exist.

Mutations within MEN1 fall into several categories. A significant number of both germline and somatic mutations are frameshift and non-sense that produce truncated proteins of various sizes [42,47]. As MENIN is a scaffold protein, N-terminal mutations of this type are predicted to severely affect protein function. A large number of missense mutations have also been identified, while InDels (insertions and deletions) and splice site variants represent the smallest fraction of mutations found.

Not all inherited mutations show the same level of cancer penetrance. Several studies have investigated the degree with which germline mutations lead to cancer phenotypes [48,49,50,51,52,53,54,55,56]. Early phylogenetic studies showed that inherited MEN1 mutations are most likely to be associated with tumors of the parathyroid, pancreas, and pituitary gland [57]. Other tumor types within additional tissues were also found at significantly lower frequencies [58]. The goal of this study was to investigate whether MEN1 germline mutations appear in cancer cells, which would suggest that they predispose patients to cancer.

The Catalogue of Somatic Mutations in Cancer (COSMIC) (https://cancer.sanger.ac.uk/cosmic) has archived MEN1 mutations that have been reported in sequenced cancer genomes [59]. To determine whether reported germline mutations have been detected or are over-represented in cancer genomes, we carried out a pan-cancer correlation study between the COSMIC data and germline mutations reported on NCBI ClinVar (https://www.ncbi.nlm.nih.gov/clinvar). From this analysis, we found that only a subset of MEN1 germline mutations are found in tumors, suggesting that the mutations likely contribute to clinical pathologies rather than to neoplasia.

## 2. Materials and Methods

An Excel file with all MEN1 COSMIC mutations was downloaded from https://cancer.sanger.ac.uk/cosmic (version 91, hg38). Patient sample data was collected from the entire COSMIC database to capture the full tumor spectrum. COSMIC deposits both primary patient data obtained from the National Institutes of Health (NIH) The Cancer Genome Atlas (TCGA) project, as well as cell line data from the Cell Lines Project. The tumor composition of the COSMIC database is accurately described at the above website and within the downloaded COSMIC mutation file for MEN1 [59]. The COSMIC file also lists PubMed IDs for most mutations. These IDs were used to extract references listed in Table S1.

Pathogenic germline mutations were first extracted from Variation Viewer (https://www.ncbi.nlm.nih.gov/variation/view) and then were manually checked against ClinVar (https://www.ncbi.nlm.nih.gov/clinvar) data to ensure that they were reported to be germline and pathogenic (e.g., reported to cause various thyroid, parathyroid, pancreatic, or pituitary syndromes). ClinVar is updated constantly with investigator submitted mutations. The data presented in this manuscript is up to date as of July 26, 2020 (Table S2). Comparisons between ClinVar and COSMIC were made manually.

Protein alignments of the various MENIN isoforms were generated using COBALT (https://www.ncbi.nlm.nih.gov/tools/cobalt/re_cobalt.cgi) [60].

Graphs and statistics were performed with SPSS (version 25) under Ohio State University (OSU) license. The statistical analysis for the mutations were performed using the online Cancer-Related Analysis of Variants Toolkit (CRAVAT) software package [61,62,63,64]. The input tables (Table S3) contained variants obtained from COSMIC (MEN1_COSMIC_CRAVAT_INPUT) or directly from the NIH TCGA repository (MEN1_TCGA_CRAVAT_INPUT). Most of the TCGA data is contained within the COSMIC file, but some values are missing as COSMIC only updates the website periodically. The Cancer-Specific High Throughput Annotation of Somatic Mutations (CHASM) and Variant Effect Scoring Tool (VEST) analysis were both performed using the default parameters of the tool. The output (Tables S4 and S5) obtained from this tool include the CHASM score and a VEST score that indicate the likelihood of a mutation being a potential cancer driver and pathogenic, respectively. In our analysis, all mutations with a Benjamini-Hochberg qVal were considered to be significant.

All diagrams and figures were made in Photoshop. Lollipop figures were made as previously described [65]. Frameshift and indel variants that appear at same position are listed only once in the main text figures (e.g., I85fs represents all I85Yfs*32, I85Sfs*33, I85Lfs*35) to decrease crowding, but the exact mutation parameters are given in Tables. If a frameshift mutation introduces a stop codon, this is indicated by an asterisk (*).

## 3. Results and Discussion

### 3.1. MEN1 and MENIN

The MEN1 gene contains 9 introns and 10 exons [43,66]. Several transcript variants and protein isoforms have been identified (Table 2) that are largely generated by alternative splicing (Figure S1) [67,68,69]. Reference genomic and amino acids sequences for alternatively spliced genes is a matter of some debate. The Human Genome Variation Society (www.hgvs.org) [70] proposes using the Locus Reference Genomic (LRG) format [71,72]. An LRG database has been compiled with the standard reference for several genes (http://www.lrg-sequence.org/search/?query=*), and the proposed standard for MEN1 is transcript variant 1 (2785bp) and protein isoform 1 (615 amino acids) (Table 2). We suggest that the LGR system of numbering amino acids be used when describing MEN1 and MENIN.

Isoform 2 has been described extensively in the literature, yet the major transcript reported on COSMIC is e1E (ENST00000337652.5) which is 3179 bases long and is translated into isoform 1 (Table 2). Both COSMIC and ClinVar databases list mutations by transcript variant. This means that, depending on which variant was analyzed, the *listed* position of the mutation (amino acid number) may differ even though the same amino acid has been changed. For example, S160 in isoform 1 is the same amino acid as S155 in isoform 2 (Figure S1), and, therefore, it may be listed as two independent mutations on COSMIC (S160 and S155) when, in fact, they are the same. To prevent over-counting such mutations and present a more unifying literature reference, we aligned protein isoforms. In the analysis presented here, mutations are only listed more than once if they were independently identified in two or more samples. The data presented here is for the 615 amino acid protein isoform 1 of MEN1. The five additional amino acids within this isoform (WSPVG) have no recorded mutations (Figure S1).

### 3.2. MEN1 Mutation Distribution

**Figure 2:**
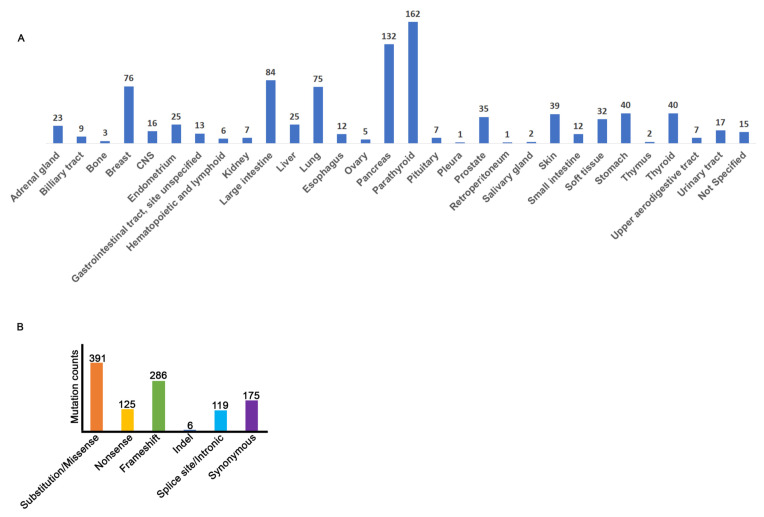
Tissue distribution of all MEN1 mutations reported on the Catalogue of Somatic Mutations in Cancer (COSMIC). (**A**) Frequency histogram of COSMIC mutations by tissue. (**B**) Distribution of COSMIC MEN1 mutations by mutation type. Splice site/intronic category also includes 5’ and 3’ untranslated regions (UTRs).

The COSMIC database reports a total of 1012 MEN1 mutations detected in 29 tumor types (Figure 2A, Table S1). For a subset of mutations, the tissue of origin was not specified, so this data was labeled as “Not Specified” (Figure 2A). As expected, a higher incidence of MEN1 mutations are found in the pancreatic and parathyroid tumors. Interestingly, a significant number of MEN1 mutations are also found in tumors of the breast, large intestine, and lung (Figure 2A). The majority of the mutations within MEN1 are substitution/missense and/or frameshifts, but non-sense, indels, splice site/intronic, and synonymous mutational events were also identified (Figure 2B, Table S1).

**Figure 3:**
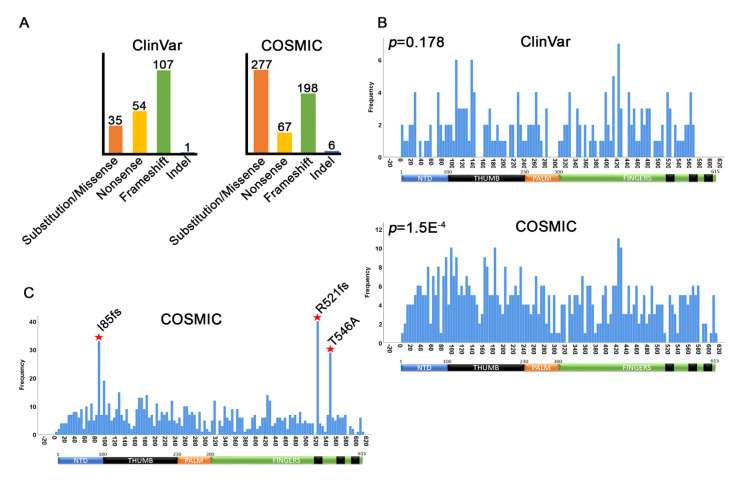
Open reading frame positions of ClinVar and COSMIC mutations. (**A**) Counts of unique residue mutation positions by mutation type. (**B**) Distribution histograms showing Kolmogorov–Smirnov P-values for uniformity. For COSMIC, only the 548 unique residues were mapped. (**C**) Histogram showing all COSMIC missense, nonsense, frameshift, and indel mutations (including duplicate) to identify hotspots. The mutations generating the three hotspots are shown.

Previous reports showed that MEN1 mutations are distributed evenly over the protein regions, suggesting that there is limited mutational selection pressure on conserved residues or domains within MEN1 [1,2]. To test whether this is true, we used the Kolmogorov–Smirnov test for uniformity (Figure S2) [73] to determine whether the germline (ClinVar) and somatic (COSMIC) mutations were evenly distributed relative to each other. For this analysis, we included only substitutions, nonsense, frameshift, and InDels as they can be directly mapped to the gene (808 out of 1202). The additional 374 mutations, which include intronic, splice site, and synonymous mutations, are discussed in later sections. From this analysis, we found that, of the 808 total COSMIC substitutions, non-sense, frameshift, and InDel mutations, 548 (67.8%) mapped to unique Open Reading Frame (ORF) positions; the remaining 32.2% mutations mapped multiple times at the same position in different samples (e.g., formed hotspots) (Figure 3A, right panel). Using ClinVar, we identified 197 germline mutations (Figure 3A, left panel, Table S2). Contrastingly, while the majority of COSMIC mutations are substitution/missense, germline mutations within the ClinVar dataset are enriched for frameshift disruptions. These results suggested that somatic and germline mutations within MEN1 may significantly differ.

The Kolmogorov–Smirnov test for uniformity (Figure S2) shows that ClinVar mutations do distribute evenly over the entire protein [74] (*p* = 0.178) (Figure 3B). Although not statistically significant, several small mutational clusters can be seen around amino acids 100, 140, and 420, respectively. In contrast, COSMIC mutations tend to cluster over key protein domains (*p* = 1.5 × 10 ^−4^) (Figure 3B). In particular, COSMIC mutations tend to cluster in the NTD and thumb domains of MEN1. Remarkably, none of these clusters correlate with the ClinVar clusters. Thus, this analysis shows that the distribution of somatic mutations of MEN1 in tumors poorly correlate with the distribution of germline mutations.

Next, we graphed all of the COSMIC mutations to identify residues that have dis-proportionately high levels of mutations. This identified three major hotspots. Two of these recurrent mutations resulted in frameshifts within MEN1 (I85fs and R521fs) and have been previously identified [1] (Figure 3C). As the I85 frameshift mutation introduces a very early termination codon, we predict that this mutation would severely limit the production of this protein isoform. The remaining recurrent mutation, T546A (T541A in isoform 2), is very close to the R521fs mutation, suggesting that disruption of the C-terminal of MEN1 may be important for tumorigenesis (Figure 3C).

Non-sense and frameshift mutations are most likely to affect critical functions of MEN1 because they cause truncations or affect the codon reading frame. While the non-sense mutations are evenly distributed for both COSMIC and ClinVar, the amino acid substitutions and frameshift mutations are not (Figure S3). Remarkably, indels are rare in ClinVar data, but they do appear in COSMIC at low frequency. ClinVar reports 20 splice site variants, of which only 7 are also detected in COSMIC tissues (Tables S1 and S2). Of the 197 total ClinVar mutations, only 78 are detected in somatic tissues. Collectively, these data suggest that the majority of germline MEN1 mutations are unlikely to contribute to the neoplasia. We discuss some of these mutations in the next sections.

Synonymous mutations were identified in both germline and somatic tissues. These mutations are often excluded as they do not alter the protein sequence. However, recent evidence shows that synonymous mutations may affect RNA splicing and mRNA stability [75]. We therefore tested the conservation of synonymous mutations within MEN1 and found no overlap between synonymous mutations identified in COSMIC or ClinVar (Tables S1 and S2).

### 3.3. Germline MEN1 Mutations in Pancreatic, Parathyroid, Thyroid, Pituitary, and Thymus Tissues

**Figure 4:**
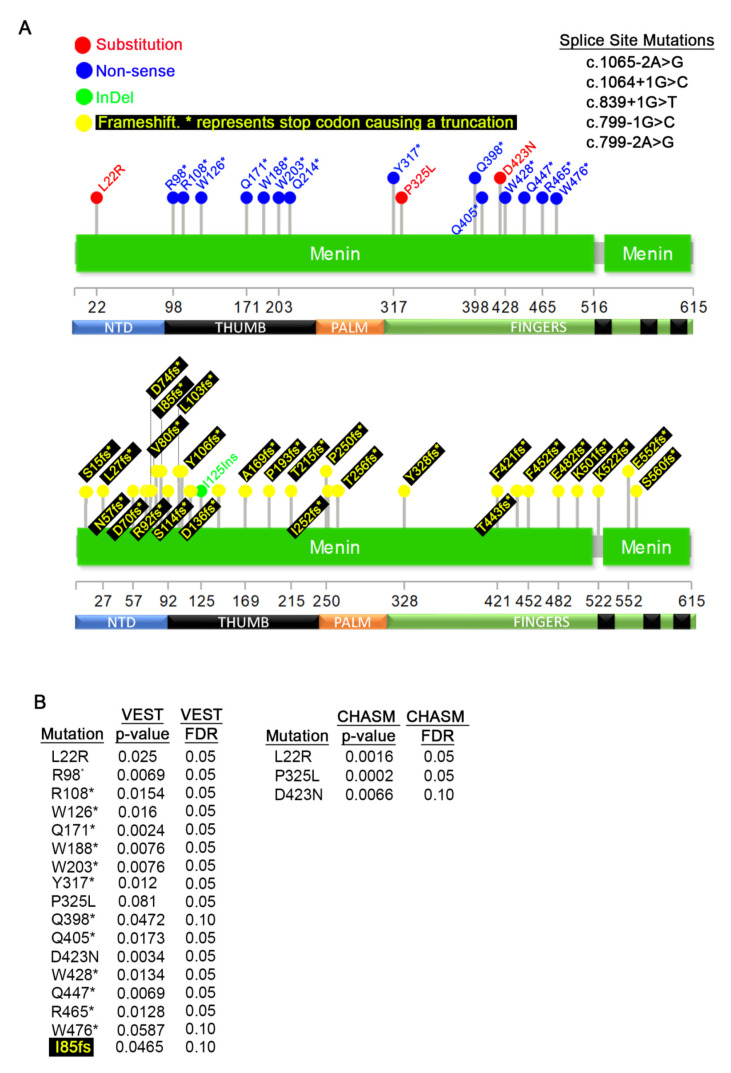
Germline mutations detected in pancreatic, parathyroid, thyroid, pituitary, and thymus cancers. (**A**) Distribution of germline mutations that appear on COSMIC. The top diagram shows missense and nonsense mutations and bottom diagram shows frameshifts and InDels. (**B**) Variant Effect Scoring Tool (VEST) and Cancer-Specific High Throughput Annotation of Somatic Mutations (CHASM) *p*-values of mutations in (**A**). For most frameshift mutations, p-values could not be calculated.

We first analyzed the MEN1 germline mutations in tissues that MEN1 mutations have been reported to contribute to cancer predisposition, such as the pancreas and the parathyroid. Less common affected tissues, such as the thyroid, thymus, and the pituitary, were also included in this analysis. From this profiling, we identified 3 amino acid substitutions, 14 non-sense mutations, 5 splice variants (all upper panel), 27 frameshift mutations (lower panel), and one indel (lower panel) with MEN1 (Figure 4A).

While non-sense and frameshift mutations are expected to produce strong phenotypes, point mutations are more likely to affect specific functions. To determine the significance of the point mutations with MEN1, we used the Cancer-Related Analysis of Variants Toolkit (CRAVAT) [61]. This biostatistics tool determines statistical significance for two parameters: 1) the probability of the alteration being a driver mutation given by the CHASM (Cancer-Specific High Throughput Annotation of Somatic Mutations) algorithm [63] and 2) the likelihood that the mutation is pathogenic determined by VEST (Variant Effect Scoring Tool) algorithm [62,64] (Tables S4 and S5). CHASM can only analyze amino acid substitutions, while VEST can analyze any mutation. For this analysis, we set a cut-off for statistical significance of a *p* < 0.05 and a False Discovery Rate (FDR) of 0.1. These metrics were selected to account for the small sample size. Using this statistical package, we found that almost all mutations within MEN1 are significant in our VEST analysis, and the two exceptions are: P325L and W476* (Figure 4B, left panel). We utilized CHASM to calculate the potential of each mutation to be a driver event and found all three point mutations, L22R, P325L, and D423N, to be statistically significant driver mutations (Figure 4B, right panel).

To examine the possible mechanisms of the mutations and the tissues of origins, we studied the existing MEN1 literature. The L22R mutation was only found in pancreatic tumors, suggesting it may have strong tissue specific effects. This mutation is predicted to limit the capacity of MEN1 to restrict the periodic expression of Cyclin B2 and partially disable cell cycle arrest at the G2/M checkpoint [76]. In addition, this mutation is required for interaction with PRMT5 [21] and, given the strong requirement of MENIN and PRMT5 in promoting pancreatic cell proliferation [77], this finding suggests that L22R may contribute to cellular growth. The P325L mutation (P320L in isoform 2) sits right at the boundary of the palm and fingers domains was only identified in parathyroid tumors [46,78]. This mutation is predicted to significantly decrease the protein stability of MENIN by targeting the protein for degradation [79]. The D423N mutant (D418N in isoform 2) is also exclusively found in parathyroid cancers [80,81]. The molecular consequence of this mutation is unexplored.

**Figure 5:**
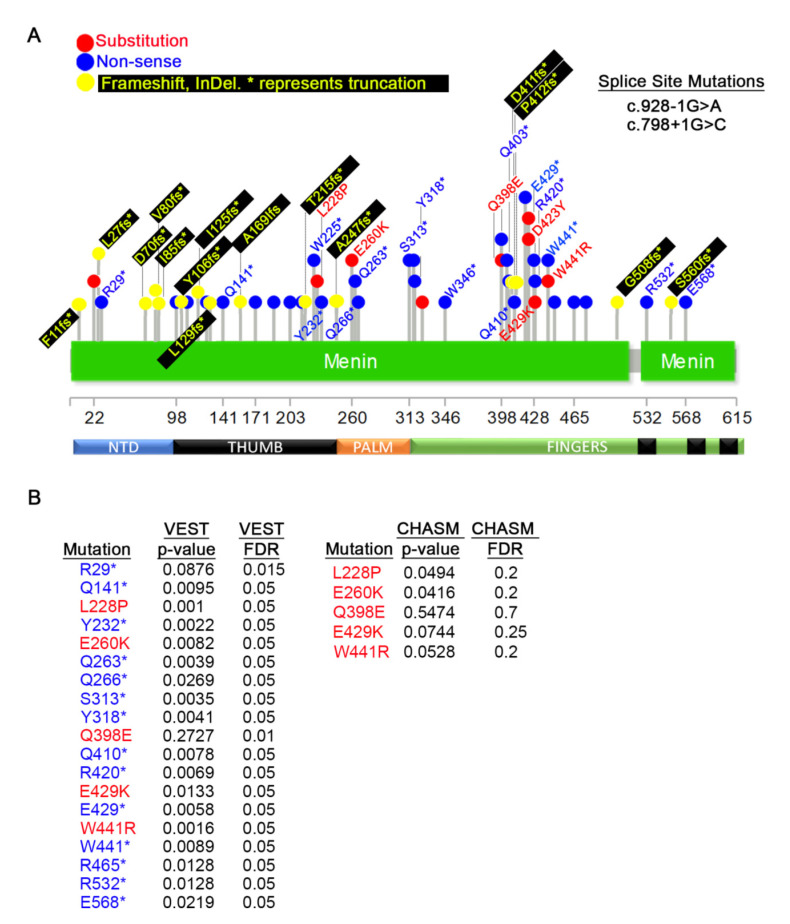
Germline mutations detected in somatic cancers. (**A**) Distribution of all germline mutations that appear on COSMIC. For completion, substitutions and non-sense mutations in pancreas, parathyroid, thyroid, pituitary, and thymus (from Figure 2A) are also shown, but they are not labeled. Only mutations with a significant VEST or CHASM *p*-value are shown. (**B**) CHASM and VEST *p*-values for the listed substitutions and non-sense mutations. Calculations for most frameshift and indels were not possible. Only I85fs values were calculated. Probability and False Discovery Rate (FDR) values for mutations already shown in Figure 4B have been excluded.

**Figure 6:**
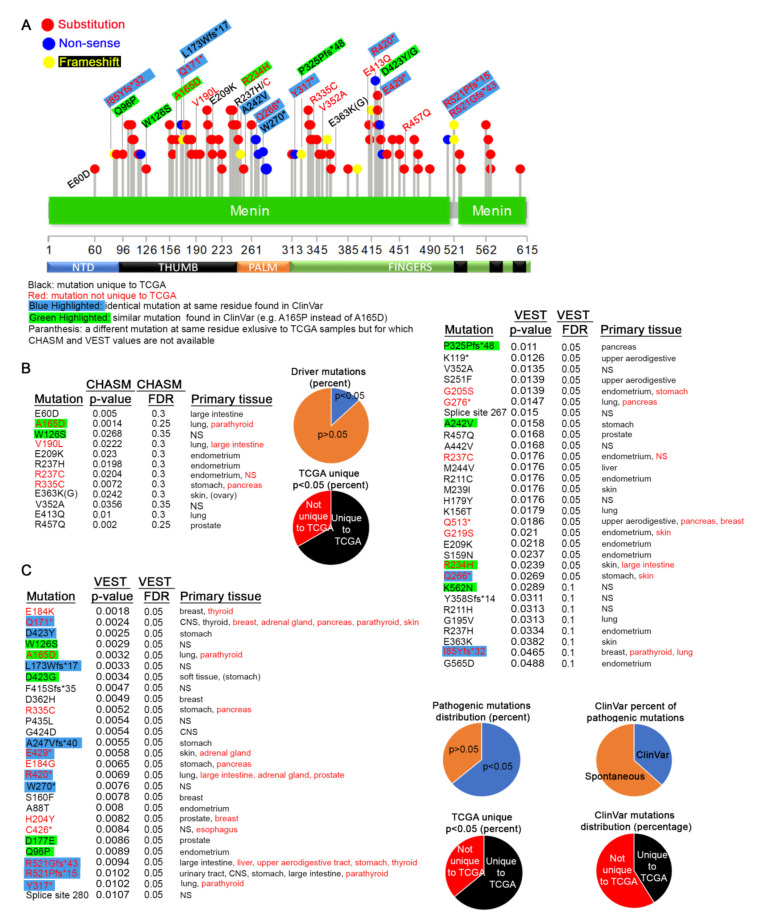
MEN1 mutations in primary cancers. (**A**) Distribution of all germline mutations that appear in TCGA samples of primary cancer sites. The legend explaining the color-coding scheme is shown below the diagram. ClinVar does not give the exact frameshift mutation, but, if a frameshift is indicated at the same residue, we assumed it to be identical. (**B,C**) CHASM, VEST *p*-values, and tissue distribution for the mutations shown in (**A**). The same color-coding scheme as in (**A**) is observed. Pie charts summarize the data in the tables.

The frameshift mutation I85fs (I85Yfs*32, I85Sfs*33, I85Lfs*35) is one of the most prevalent germline mutations both in parathyroid, thyroid, and pancreatic cancers, as well as other cancer types (also see Figure 5 and Figure 6) [1,41,82,83,84,85,86,87,88,89,90,91,92,93,94,95]. Although this mutation has been previously shown to be a hotspot, only one mutational event was identified, c.249_252delGTCT [1]. In our analysis, we found that this position is characterized by at least three types of frameshifts caused by deletions and insertions (Table S1). Each of these frameshifts are predicted to terminate the translation of the MEN1 RNA within 32–35 amino acids, thus producing a truncated and/or unstable protein. Considering that several frameshift mutations occur within the same region, it is not immediately clear why the I85fs mutation should occur at higher frequency than the others. This mutation occurs within the center of exon 2 within a region that is characterized by the repeated amino acids Ile-Ile-Ala-Ala (atc atc cgc cgc), and we speculate that this may cause higher rates of DNA polymerase slippage. At least one report shows that no minimum nucleotide repeats are required for DNA polymerase slippage [96].

### 3.4. Germline MEN1 Mutations Penetrant in other Cancers

Next, we investigated the frequency of germline MEN1 mutations in all tumor types. From this analysis, 44 germline mutations were identified (Figure 5A). For comparison and visualization, we also include mutations shown in Figure 4A, and only mutations that have significant CHASM or VEST p-values are labeled. The CRAVAT analysis shows that most mutations are likely to be pathogenic (Figure 5B, left panel). In contrast to the mutations enriched in tumors from MEN1 sensitive tissues (parathyroid, pancreas), none of the point mutations found in other tumors types are driver mutations based on our CHASM criteria (Figure 5B, right panel). Although L228P and E260K both have CHASM *p*-values just below 0.05 (0.0494 and 0.0416, respectively), their FDR scores are well outside statistical significance.

Several highly enriched and significant truncating mutations appear within the thumb region of MEN1 (aa 98-188). The Q171* (VEST *p*-value = 0.0024) non-sense mutation is present in nine COSMIC samples (Figure 4A, Figure 5A) and is also detected in primary cancer tissues (see Figure 6A). In addition to our analysis, this mutation has also been identified in a metastatic cancer screen [82], two breast cancer mutation analysis studies [97,98], and adrenal cortical adenoma [99]. A frameshift mutation (Q171Rfs*19) has also been identified in parathyroid tumors [100]. The R98* mutation (VEST *p*-value = 0.0069) was detected in 7 samples in our analysis and has been identified in several tumors [82,84,101,102,103]. A point mutant (R98Q) and a frameshift variant (R98Efs*21) has also been detected [104]. The W126* non-sense mutation (VEST *p*-value = 0.0024) that is positioned closely to the highly mutated Q171 residue is also reported five times in COSMIC data. It, too, has been identified in metastatic [82] and breast [105] cancers, as well as pancreatic [83] and adrenal [106] tumors. One frameshift mutation at this position (W126fs*23) was identified in metastatic and thyroid cancers [82,107], and a W126G point mutation at this location has been reported [85]. Q141* (VEST *p*-value = 0.0095) and R108* (VEST *p*-value = 0.0154) are two other truncations in this region that appear with higher frequency in various cancers [82,89,108,109,110,111,112]. These examples suggest that our analysis has identified recurrent and important MEN1 alleles that contribute to the tumorigenic process. Why these five truncating mutations within the same region should be more penetrant than others remains unknown, but it suggests that mutations in this region may destabilize broader functions of MENIN that manifest in multiple cancer types.

### 3.5. MEN1 Mutations in Primary Tumors

COSMIC provides an exhaustive collection of data from multiple sources. This includes reports from both TCGA samples that are derived from patient tumor samples, as well as non-TCGA resources, which may include cultured cell lines. We next wanted to focus solely on the mutational spectrum of MEN1 in patient tumors. For this, we accessed reported MEN1 mutations directly from the NIH TCGA website. These data include a few other mutations not reported in COSMIC version 91. This analysis identified 91 mutations within the MEN1 gene from TCGA primary tissues (Figure 6A).

We next tested the potential for each of these events to be driver mutations using CHASM, and, although some presented *p*-values below 0.05, we consider them not to be significant based on their high FDR scores (Figure 6B). Of these, only one similar mutation at A165 (CHASM *p*-value = 0.014, VEST *p*-value = 0.0032) was also found in the germline dataset. The W126S mutation does not have an FDR score below 0.3. In contrast, 57 pathogenic mutations were found to be statistically significant using VEST (Figure 6C). Unsurprisingly, most of the other mutations with VEST *p*-value below 0.05 are also unique to TCGA (Figure 6C). A small subset of these mutations has been reported as rare events in non-COSMIC datasets. These data strongly suggest that MEN1 mutations detected in patient tumor samples most likely appear spontaneously and are somatic in nature.

The two frameshift mutations forming the hotspot at position R521 (R521Gfs*43 VEST *p* = 0.0094 and R521Pfs*15 VEST *p* = 0.0102) (Figure 3C) [1] have also been found in germline tissues and have been detected 25 times in several cancers, including thyroid [113,114], parathyroid [41,100,102,103], colorectal [115,116,117,118], pancreatic [119], gastric [120,121], and lung [122], as well as other metastatic cancers [82] (Tables S1 and S2). Two other mutations at this locus (R521Q and R521W) that do not have significant CHASM or VEST value in our study have also been identified. R521 is positioned within a finger region between NLS1 and NLS3, and, although the exact role of these mutations is not known, the R521Gfs*43 likely disrupts the function of NLS3. The neighboring T546A (T541A in isoform 2) hotspot (CHASM *p* = 0.004, VEST *p* = 0.8887) appears in 24 samples [123,124] (Tables S1 and S2). This mutation, which has also been detected in germline tissues, appears to affect apoptosis [125]. The I85 hotspot is generated mainly by the I85Yfs*32, I85Sfs*33, I85Lfs*35, and I85V mutations and is discussed in the previous sections.

## 4. Conclusions

In this study, we showed that only a fraction of inherited MEN1 aberrations appear as somatic mutations in tumors. Although germline MEN1 mutations contribute to the clinical pathologies of the parathyroid, thyroid, and pancreatic syndromes, we find that very few contribute to neoplasia and cancer. As neither COSMIC nor TCGA contains inherited MEN1 mutant patient data, our analysis is limited to making predictions on the contribution of these events to tumorigenesis. However, as this is a pan cancer analysis it suggests that, if MEN1 germline mutations significantly contributed to cancer phenotypes, they would be detectable in various patient samples. As our analysis shows that only a small fraction of the mutations appears in tumors, we speculate that many MEN1 germline mutations do not drive neoplasia. We further show that, of the germline mutations that do appear in tumors, only a small fraction has the potential to be pathogenic or driver mutations. The clinical value of this data is to highlight the need for somatic testing of cancer patients with MEN1 disruptions, rather than, or at least in addition to, pedigree studies that focus on cancer pre-disposition syndromes.

## Figures and Tables

**Table 1:** Coordinates and functions of MEN1 domains and interacting proteins.

Gene or Region	MEN1 Coordinates (Amino Acids)	Function
*MEN1 Domains*		
NTD	1–100	Crystal structure identified domain
Thumb	101–1230	Crystal structure identified domain
Palm	231–1300	Crystal structure identified domain
Fingers	301–1305	Crystal structure identified domain
NLS1	479–1497	Nuclear localization sequence
NLS2	588–1608	Nuclear localization sequence
NLS3	546–1672	Nuclear localization sequence
NES1	33–151	Nuclear exit sequence
NES2	253–1267	Nuclear exit sequence
*MEN1 Interacting Proteins*		
KMTA2 (MLL1)	~230–1300	Mixed lineage leukemia
LEDGF	~1–1100	Chromatin associated factor, required for MLL oncogenic transformation
JunD	1–140, 323–1448	Transcriptional activator, subunit of AP-1 transcription complex
HLXB9	41–1177, 323?	Homeobox gene involved in pancreas development, neural motor protein
SMAD3	41–1278, 477–1615	TGFB signaling pathway, cell proliferation, transcriptional regulation
MYC	360-615 (fingers), maybe NTD	Transcriptional regulation, cell cycle, apoptosis, cellular transformation
PEM (mouse)	278–1476	Homeobox gene, embryonic and placenta expression
NF-kB subunits	305–1476	Transcriptional regulators, inflammation, immune response, cell proliferation
SIRT1	305–1476	Sirtuitin, gene silencing
SIN3A	295–1450	Gene expression regulator, embryogenesis, cell proliferation, senescence
HDAC1	295–1450	Histone deacetylase, transcriptional regulator, cell proliferation and differentiation
PRMT5	L22, A242	Arginine methyltransferase, transcriptional regulation, DNA damage repair
FANCD2	219–1395	Fancomi anemia complex subunit, DNA damage repair
RPA2	1–140, 286–1448	Replication Protein A subunit

**Table 2:** MEN1 transcript variants and their corresponding MENIN protein isoforms.

^1^ Transcript Variant	Transcript Size (Bases)	Protein Isoform	Protein Size (Amino Acids)
1	2785	^2^ 1	615
e1B	2748	1	615
e1C	2736	1	615
e1D	3712	1	615
e1E	3179	1	615
e1F1	3015	1	615
2	2770	^3^ 2	610
3	2828	3	652
4	2712	2	610
5	2702	2	610
6	2960	2	610
7	2855	4	575
8	2609	4	575
^4^ MEN1-207	3150	2	610
^5^ MEN1-205	2868	?	555
X1	3629	X1	657
X2	3629	3	652
^6^ Isoform alignment schematic. 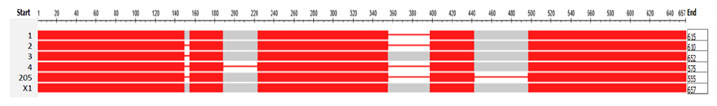			

^1^ The names and sizes of variants and isoforms are from NCBI. ^2^ This is one of the two major isoforms of MENIN. ^3^ This is another of the two major isoforms of MENIN. ^4^ This variant refers to transcript ENST0000377326.7 but does not have a corresponding variant name on NCBI. It is listed as Men1-207 on Ensembl. ^5^ This variant refers to transcript ENST0000377316.6 but does not have a corresponding variant name on NCBI. It is listed as MEN1-205 on Ensemble. ^6^ This is a schematic diagram generated by COBALT showing the size differences of the various MENIN isoforms. For amino acid alignment, please see Figure S1.

## Supplementary Materials

The following are available online at https://www.mdpi.com/2072-6694/12/9/2616/s1, Figure S1: Amino acid sequence alignment of various MENIN isoforms, Figure S2: Example of the Kolmogorov-Smirnov uniformity test statistic, Figure S3: Distribution of ClinVar and COSMIC mutations by type, Table S1: Summary of MEN1 mutations reported on COSMIC, Table S2: ClinVar confirmed pathogenic mutations, Table S3: CRAVAT input values, Table S4: CRAVAT output non-TCGA values, Table S5: CRAVAT output TCGA values.
