# Supplementary material for: Comprehensive Analysis of MEN1 Mutations and Their Role in Cancer
Source: Cancers (Basel). 2020 Sep 14;12(9):2616. doi: 10.3390/cancers12092616 (PMC7565326; doi:10.3390/cancers12092616)
Supplement: Supplementary file 1 [file cancers-12-02616-s001.zip › cancers-901091-final check-supplementary/cancers-901096-layout-Supplementary.docx]

Supplementary Materials

Comprehensive Analysis of MEN1 Mutations and Their Role in Cancer

Devi D. Nelakurti, Amrit L. Pappula, Swetha Rajasekaran, Wayne O. Miles and Ruben C. Petreaca


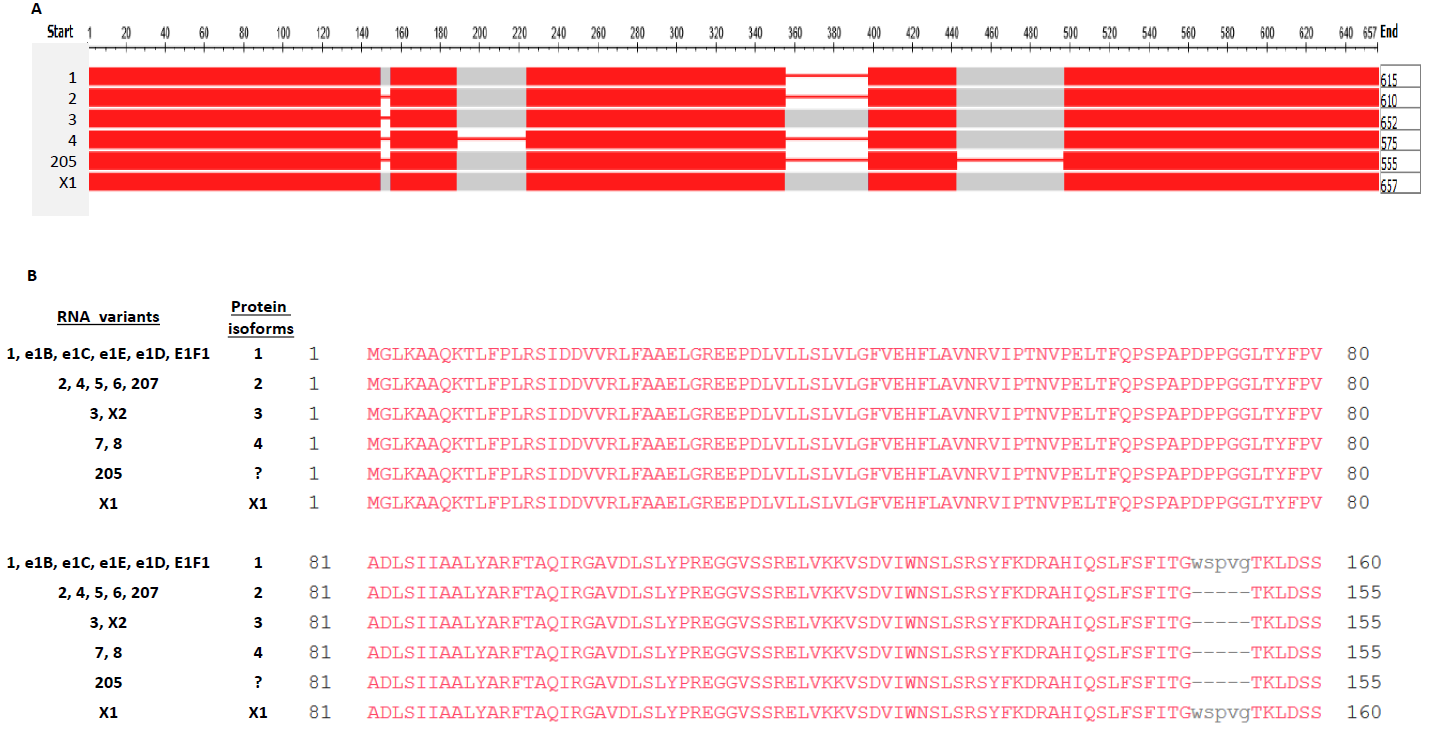


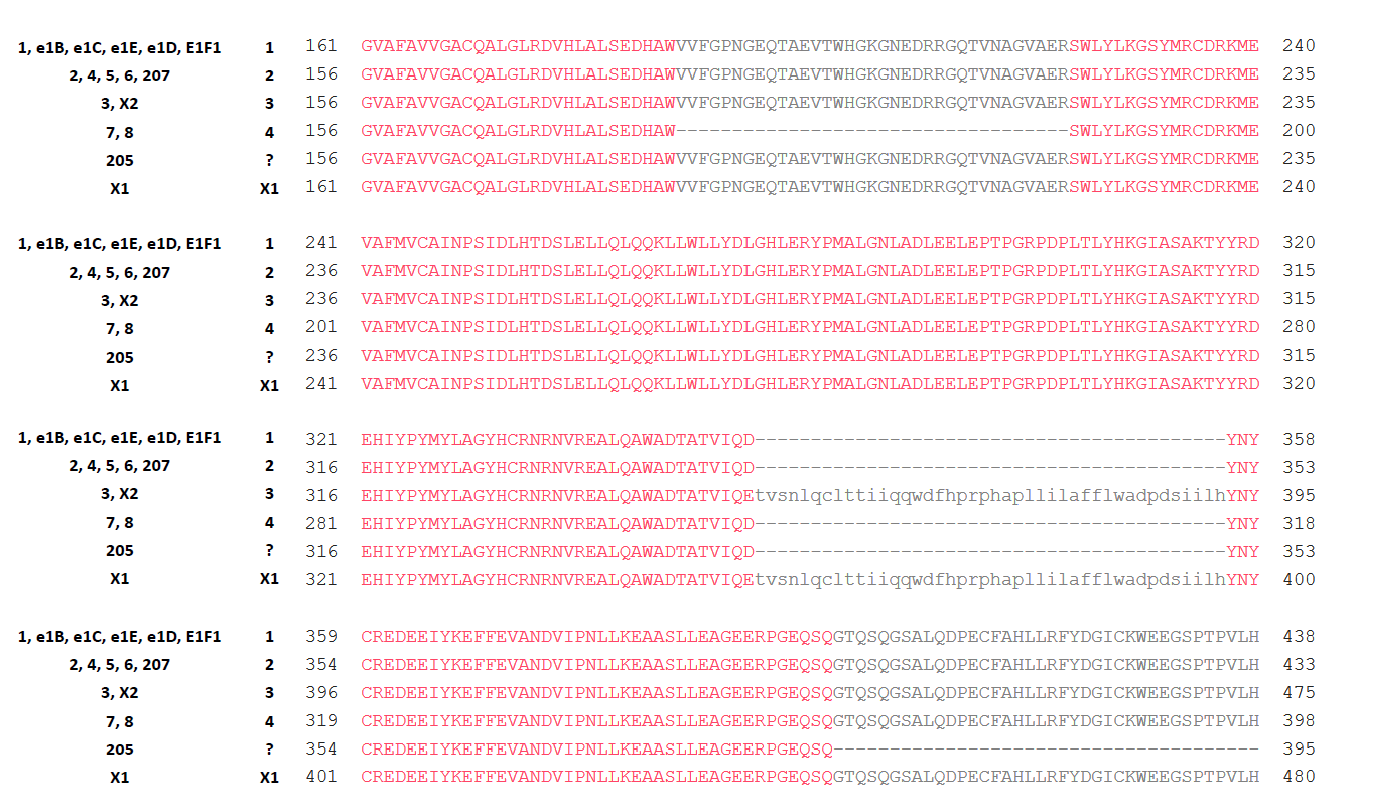


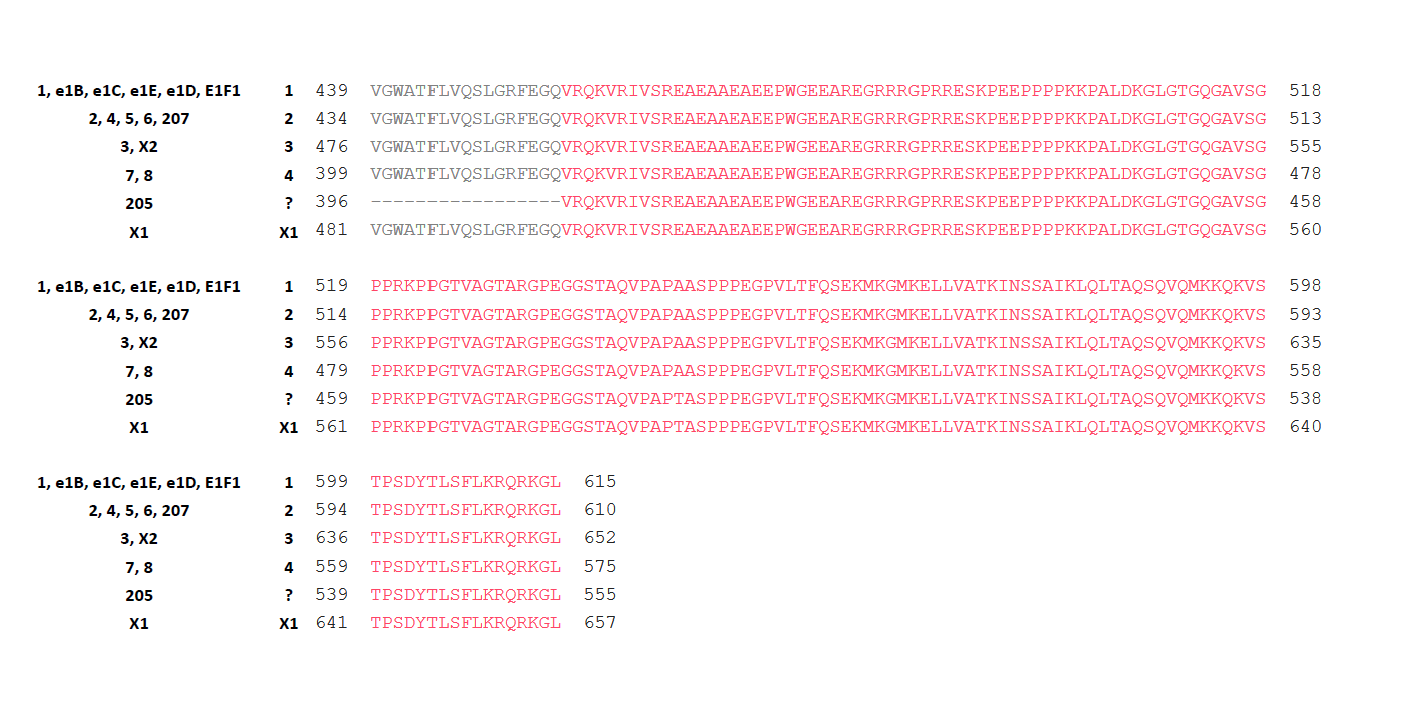


**Figure S1.** Amino acid sequence alignment of various MENIN isoforms.

**
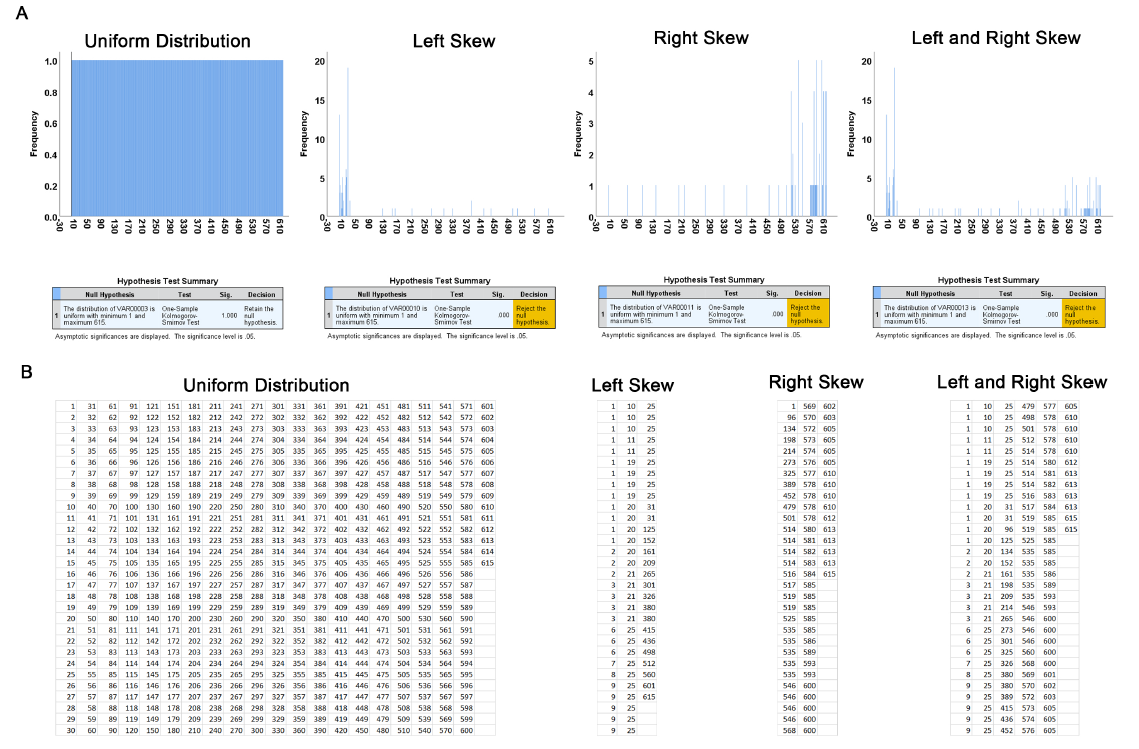
**

**Figure S2.** Example of the Kolmogorov-Smirnov uniformity test statistic.


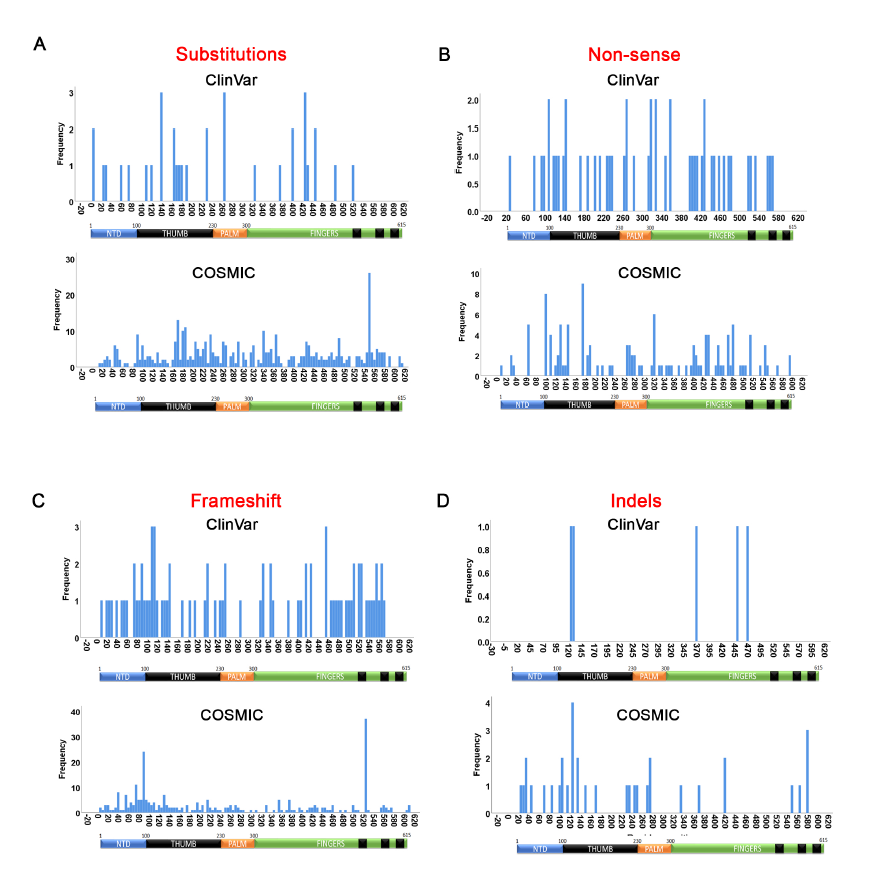


**Figure S3.** Distribution of ClinVar and COSMIC mutations by type.

**Table S1.** Summary of MEN1 mutations reported on COSMIC. The references queried by COSMIC for some of the mutations are shown. For some, COSMIC does not provide references. For more information please access the COSMIC file as described in Materials and Methods.

| **Tissue** | **Mutation CDS** | | | **Reference** |
| --- | --- | --- | --- | --- |
| Adrenal Gland | p.G42D, p.D102Pfs*12, p.E109*, p.R176Q, p.R234C, p.L275M, p.G276A, p.H277R, p.R335H, p.E383*, p.R420*, p.E429*, p.R451C, p.E453*, p.T557S, p.T604Sfs*79 | | | [1–8] |
| Biliary Tract | p.A163T, p.R360W, p.A373V, p.E473Kfs*85, p.G525Rfs*11 | | | [9–12] |
| Bone | p.I85Sfs*33, p.E295Q, p.E371Kfs*2 | | | [11,13,14] |
| Breast | p.L36F, p.F43_F47del, p.D70Afs*45, p.I85S/Yfs*33, p.L89R, p.Q96*, p.L103_S104insC, p.L103dup, p.R108P, p.K120*, p.L129Pfs*25, p.S160F, C170Y, p.E184K, p.E200K, p.H204Y, p.V220M, p.S258L, p.K267Sfs*19, p.L272R, p.A289E, p.R319W, p.T530I, p.D362H, p.Q344Gfs*25, p.R490Q, p.E491*, p.S606C, p.Q513*, p.R521Pfs*15, p.P534A, p.P554A, p.K562M, p.K562del, p.M563I, p.K567Sfs*24, p.L570P/Q, p.S606Vfs*78, p.L605V, p.S606Y | | | [11,15–21] |
| Central Nervous System | p.G99S, p.L129F, p.V167L, p.G205D, p.R337H, p.A408T, p.G424D, p.T443I, p.R521Pfs*15 | | | [11,22,23] |
| Endometrium | p.A88T, p.Y90H, p.Q96P, p.G110W, p.S159N, p.R211C, p.G219S, p.G205S, p.E209K, p.R223Q, p.R237C/H, p.R319W, p.R337H, p.R340W, p.A385V, p.L419_G431delinsH, p.K427E, p.G565D, p.E568G, p.R612H | | | [11] |
| Esophagus | p.F47I, p.R337C, p.C426*, p.R489W, p.Q541*, p.F558L | | | [11,24] |
| Gastrointestinal Tract | p.A350Pfs*23 | | | [25] |
| Gastrointestinal Tract (Site Indeterminate) | p.G42S, p.S66_P67del, p.K120del, p.F134Lfs*20, p.I140Tfs*14, p.E184*, p.G213Afs*16, p.G230Dfs*56, p.W270Cfs*16, p.A547_L556del, p.T539M | | | [26–28] |
| Hematopoetic and Lymphoid | p.T546A | | | N/A |
| Kidney | p.G161C, p.G508Afs*56 | | | [29] |
| Large Intestine | p.E60D, p.V80L, p.I97L, p.P107R, p.K120del, p.V167F, p.V190L, p.R223W, p.M233T, p.R234H, p.G230R, p.L261I, p.Q263H, p.H277Y, p.G299D, p.K315N, p.R319W, p.R335H, p.R340Q , p.F369L, p.E392K, p.P395L, p.E413A, p.R420*, p.P433H, p.P433L, p.V439M, p.P475L, p.R461C/H/Q, p.R484W, p.R485Q , p.R489Q, p.R490Q, p.L509M, p.R521Pfs*15, p.R521Gfs*43, p.Q541H, p.P543S, p.M566I, p.S577L, p.S578_A579del, p.S588L | | | [11,30–37] |
| Liver | p.K8*, p.S38F, p.R52G, p.T62Sfs*59, p.F164S, p.E184V, p.A218D, p.M244V, p.W346G, p.R360W, p.R521Gfs*43, p.A544E, p.Q559Pfs*3, p.T573N | | | [11] |
| Lung | p.K8Nfs*107, p.D33Afs*81, p.E45V, p.E45Gfs*, p.N51I, p.E60*, p.S84_Y90del, p.I85V, p.I85S/Yfs*33, p.L89R, p.L103M, p.Y106Rfs*7, p.R108L, p.G110A, 72, p.R131C, p.R137G, p.L143Sfs*11, p.K156T, p.A163D, p.A165D, p.V167F, p.A169S, p.D177V, p.A181T, p.S183C, p.V190L, p.G195V, p.E196D, p.E196Dfs*3, p.A221T, p.E222V, p.R223W, p.Y232dup, p.G276*, p.L288P, p.R300L, p.G310D, p.S313L, p.Y317*, p.C334F, p.Y366C, p.N379Tfs*3, p.A385D, p.P412Rfs*41, p.E413Q, p.R420*, p.G440Afs*10, p.A442D, p.V456Cfs*7, p.A470V, p.R485W, p.R489L, p.S560Rfs*2, p.A579S, p.G508D, p.R521Pfs*15, p.G525V, p.P551L, p.S606Vfs*78 | | | [11,38–51] |
| Ovary | p.N217T, p.Q354E, p.E363G, p.S588L | | | [52–54] |
| Pancreas | p.M1_?37, p.L13Q, p.L13Tfs*86, p.D17Afs*101, p.R29Q, p.V53Sfs*66, p.L83Rfs*36, p.E30*, p.P32R, p.V40M, p.E45D, p.H46P, p.I54Pfs*63, p.E60Sfs*59, p.Q64*, p.D70Efs*44, p.D70Tfs*49, p.P72Afs*45, p.G73Vfs*40, p.I85Sfs*33, p.I85Lfs*35, p.L89R, p.A95Pfs*24, p.V101Rfs*16, p.L105Pfs*12, p.G111Afs*68, p.K120del, p.I125Mfs*59, p.W126G, p.Q141R, p.S145R, p.F146Lfs*39, p.I147del, p.D158Tfs*32, p.V162Gfs*22, p.V167C, p.A169V/E, p.G174C, p.R176Q, p.L180P, p.E184*, p.S183V/Y, p.W188R, p.N194Mfs*35, p.E200Qfs*2, p.H204Q, p.R223Efs*13, p.L226_L228del, p.Y227Tfs*2, p.G230V, p.M239Gfs*14, p.E240del, p.P250Lfs*36, p.I252Lfs*34, p.E260*, p.L261F, p.L272Qfs*42, p.G276*, p.L278Gfs*43, p.L285*, p.A289V, p.P325R, p.Y326*, p.Y326Tfs*47, p.Y328Tfs*45, p.L343P, p.A350Pfs*23, p.V352Sfs*21, p.R335C/P, p.Y358C, p.E392Rfs*58, p.E397*, p.C426Afs*24, p.H438Lfs*12, p.L409Sfs*41, p.L419P, p.W428R, p.E496*, p.Q513*, p.A540V, p.S548*, p.Q541*, p.T546A, p.S560Rfs*3, p.K574Rfs*19, p.I575_A579delinsT | | | [11,25,27,55–67] |
| Parathyroid | p.K4Hfs*101, p.R14Afs*105, p.S15Cfs*99, p.L22_P59del, p.E26K, p.L27Gfs*84, p.G28_L39del, p.E30_F47delinsD, p.E31del, p.P32R, p.S38Wfs*75, p.S38Ffs*79, p.V40Pfs*63, p.G42S p.E45D, p.N51Tfs*65, p.N51Qfs*66, p.N51Tfs*68, p.I54Mfs*63, p.N57Ffs*61, p.E60, p.T62Hfs*55, p.P65Rfs*43, p.G74Afs*45, p.F63Lfs*54, p.A68Gfs*49, p.A68Pfs*50, p.D70Pfs*42, p.D70Rfs*47, p.D70Tfs*49, p.Y77Sfs*42, p.A81Gfs*36, p.D82Tfs*37, p.I85S/Yfs*33, p.I86Mfs*32, p.L89R, p.R92Sfs*26, p.I97Mfs*19, p.I97_L103del, p.R98Efs*21, p.D102Sfs*10, p.S114_W126delinsR, p.R115Efs*65, p.R115Vfs*4, p.K120del, p.V121D, p.V121Gfs*65, p.S122Cfs*61, p.I125Pfs*22, p.Y133Lfs*52, p.Y133_L143del, p.F134Sfs*20, p.D136Ifs*18, p.H139Y, p.W150Afs*3, p.V162Cfs*23, pQ171*, p.Q171Rfs*19, p.F146Sfs*8, p.G154Afs*36, p.A165D, p.V167F, p.A169_C170delinsG, p.A169D, p.A172Sfs*10, p.A181Vfs*5, p.D185V, p.V189*, p.P193T, p.P193Sfs*33, p.T202Nfs*21, p.H204Qfs*25, p.R211Pfs*9, p.T215Sfs*13, p.V216F, p.K229Ifs*6, p.E240K, p.C246Pfs*39, p.A247_I252del, p.T256Pfs*30, p.D257Gfs*15, p.S258L, p.L259Sfs*23, p.K267_Y273del, p.W270_L272del, p.Y273_R280delins*, p.Y273*, p.M283Rfs*30, p.A289P, p.P296A, p.L306Sfs*67, p.Y317*, p.Y332*, p.Y332_H333insS, p.T351Lfs*22, p.F369_V372delinsL, p.F369*, p.K367Lfs*2, p.L381Rfs*68, p.E392Rfs*22, p.E393*, p.G396Rfs*58, p.A416Pfs*34, p.L419_E430del, p.F421Lfs*26, p.F421Sfs*29, p.G424Afs*26, p.H438Rfs*12, p.T443Kfs*14, p.S448P, p.F452Lfs*11, p.Q458*, p.K459Rfs*4, p.K459Sfs*76, p.E474*, p.E482Dfs*79, p.K501Qfs*35, p.R521Pfs*15, p.Q541*, p.P551Rfs*13, p.E552Tfs*15, p.P55Sfs*58, p.F558Sfs*6, p.M566T, p.T573Dfs*27, p.S578_A579del, p.T585R | | | [8,11,51,61,68–87] |
| Pleura | p.V189Gfs*5 | | | [11] |
| Pituitary | p.V80Wfs*39, p.Y90Mfs*29, p.H179P, p.F415L, p.K507M | | | [66,88–90] |
| Prostate | p.R98Q, p.R176Q, p.D177E, p.H204Y, p.G213A, p.V245L, p.S258L, p.R420*, p.Y422N, p.T434M, p.R451H, p.R457Q , p.P475L, p.G487V, p.P500S, p.P545R, p.P550T | | | [11,91–95] |
| Retroperitoneum | p.D411Afs*39 | | | [11,96] |
| Salivary Glands | p.F11Lfs*106, p.Y106Efs*11, p.I575M | | | [97] |
| Skin | p.A163V, p.L175F, p.L180F, p.T202I, p.G205C, p.G219S, p.R234H, p.M239I, p.E292*, p.D348N, p.T351I, p.Q354*, p.R360L/W, p.E363K, p.G396V, p.E429*, p.W428C/L, p.R451C, p.R481W, p.R484W, p.S492F, p.E495K, p.S606Ffs*77 | | | [11,63,98–102] |
| Small Intestine | p.L36H, p.I85Sfs*33, p.I86F, p.L117V, p.G213Afs*16, p.A350Pfs*23, p.A373Mfs*7, p.A470T, p.A528T | | | [11,25,57,103–105] |
| Soft Tissue | p.D17Efs*102, p.Q64*, p.P67L, p.I85Sfs*33, p.K135*, p.R176Q, p.E364K, p.D423G, p.T546A | | | [11,85,106–108] |
| Stomach | p.V35M, p.Y77Sfs*42, p.I85Sfs*33, p.G111S, p.E184G, p.G205S, p.A242V, p.A247Vfs*40, p.S258L, p.A284T, p.P296S, p.R335C, p.G391D, p.P412T, p.W428L, p.R457W, p.R489Q, p.K501R, p.A504Gfs*22, p.R521Gfs*43, p.R521Pfs*15, p.R521Q/W | | | [1,11,67,109–112] |
| Thymus | p.I86Sfs*34, p.L445Cfs*8 | | | [113] |
| Thyroid | p.E26K, p.F23C, p.Q64*, p.R92H, p.E184K, p.I85S/Yfs*33, p.Y106Cfs*5, p.W126Afs*23, p.S142P, p.T215Sfs*13, p.L288P, p.P298Gfs*73, p.E341*, p.V352D, p.I365M, p.F421L, p.K427Nfs*23, p.A469V, p.P498Rfs*66, p.P497Efs*34, p.R521Gfs*43, p.K522Sfs*42, p.T526S, p.M592* | | | [11,114–118] |
| Upper Aerodigestive Tract | p.K119*, p.S251F, p.A347V, p.A416T, p.Q513* p.R521Gfs*43 | | | [11,119,120] |
| Urinary Tract | p.S15C, p.F23Lfs*87, p.D33Y, p.R115Gfs*67, p.Y133*, p.S313L, p.A314P, p.R340Q, p.E371Mfs*8, p.R420Q, p.R521Pfs*15, p.A579T, p.Q591* | | | [11] |
| Not Specified | p.S38F, p.L83Pfs*28, p.E116Q, p.I125Mfs*59, p.F164C, p.R237C, p.V245L, p.L261Afs*14, p.S258L, p.A373Mfs*7, p.S548L | | | [11,57,103,121] |
| **Tissue** | **Splice site and intronic mutations** | | | **Reference** |
| Adrenal Gland | c.1366-4C>T, c.1365+1G>T | | | [3,7] |
| Biliary Tract | c.461-60G>A, c.670-5del, c.461-1G>A, c.670-43G>A | | | [9,11] |
| Breast | c.*550C>G, c.1064+25G>A, c.928-1G>A, c.839+2T>G, c.*73C>G, c.*241A>C, c.*225C>G | | | [122] |
| Central Nervous System | c.460+5G>A | | | [22] |
| Endometrium | c.*770G>T, c.*805T>G, c.*42G>A | | | N/A |
| Esophagus | c.798+14C>T, c.461-584G>A, c.927+102T>A, c.927+101G>T, c.461-585C>T, c.928-215C>G | | | N/A |
| Gastrointestinal Tract (site indeterminate) | c.1065-5_1066del | | | [28] |
| Hematopoietic and Lymphoid | c.1065-71G>A, c.460+701G>A, c.-23-55A>T, c.-471A>T, c.460+717G>C | | | [11] |
| Kidney | c.-417C>T, c.1201-1G>T, c.*85C>T, c.669+33C>T | | | N/A |
| Large Intestine | c.669+18del, c.669+23T>G, c.461-253G>T, c.1065-202A>T, c.460+704G>A, c.1065-71G>A, c.*28G>A, c.670-5del, c.928-146G>A, c.1064+115A>G, c.1065-71G>A, c.461-51C>T | | | [31,32,35] |
| Liver | c.798+1G>C, c.*83C>A, c.670-1G>A, c.670-1G>A, c.460+683T>A, c.-23-327G>T, c.-24+173A>T | | | [11] |
| Lung | c.669+3A>G, c.669+1G>A, c.460+168G>A, c.670-2A>C, c.*126C>G, c.927+255C>A, c.1366-67C>A | | | [11,43,48] |
| Ovary | c.-320C>G | | |  |
| Pancreas | c.839+1G>T, c.928-115_940del, c.412del, c.1064+1G>C, c.1366-1G>A, c.799-2A>G, c.669+1G>T, c.799-1G>T, c.1065-1G>A, c.927+1G>A, c.840-2A>G, c.669+2T>A, c.787_798+3del | | | [11,25,55,58,123] |
| Parathyroid | c.799-1G>C, c.839+2T>G, c.927+2T>A, c.-19_52del, c.1366-1_1372del, c.839+1G>T, c.839+1G>A, c.669+3A>G, c.839+2T>G, c.927+1del | | | [8,51,61,68–70,72,86] |
| Pituitary | c.822_839+4del, c.1065-2A>G | | | [124,125] |
| Prostate | c.1065-71G>A, c.-282T>G, c.460+663A>C, c.927+1G>T, c.460+717G>C, c.-20G>A | | | [94,95] |
| Skin | c.*90G>T, c.1064+3G>T, c.*45C>A, c.1065-100T>C, c.-23-1G>A | | | [11,108] |
| Soft Tissue | c.1064+9C>T | | | N/A |
| Stomach | c.670-5del, c.670-5dup, c.1365+54G>A, c.460+34dup, c.669+18del | | | N/A |
| Thyroid | c.839+1G>A, c.840-2A>T | | | [114] |
| **Tissue** | **Silent mutations** | | | **Reference** |
| Breast | p.D423=, p.L129=, p.R108=, p.S598=, p.T573= | | | [126] |
| Central Nervous System | p.F558=, p.H308=, p.L570=, p.R223=, p.S160= | | | [22,127,128] |
| Cervix | p.F144= | | |  |
| Endometrium | p.A242=, p.G565=, p.K119=, p.S407= | | |  |
| Esophagus | p.C246=, p.L605= | | | [129–131] |
| Hematopoetic and lymphoid | p.A181=, p.D423=, p.T434= | | |  |
| Kidney | p.L143= | | |  |
| Large Intestine | p.A470=, p.D423=, p.R300=, p.S160=, p.T349=, p.D362=, p.E60=, p.P550=, p.T585= | | | [32,33] |
| Liver | p.D423=, p.G424=, p.H333=, p.I248=, p.L329=, p.R211=, p.S231= | | |  |
| Lung | p.A408=, p.A469=, p.D423=, p.G525=, p.R610=, p.S160=, p.T434= | | | [41,132,133] |
| Ovary | p.H333= | | |  |
| Pancreas | p.A470= | | |  |
| Prostate | p.F144=, p.G213= | | |  |
| Skin | p.A347=, p.E200=, p.F607=, p.L175=, p.L275=, p.L304=, p.L615=, p.Q171=, p.Q458= | | | [98,101,102,133] |
| Soft Tissue | p.L117=, p.S145= | | | [108,134] |
| Stomach | p.A469=, p.A470=, p.P475= | | |  |
| Thyroid | p.D423=, p.K501= | | |  |
| Upper Aerodigestive Tract | p.A469=, p.I54= | | |  |
| Urinary Tract | p.A408=, p.L129=, p.L228=, p.L608=, p.S577= | | |  |
| Not Specified | p.I97= | | | [135] |
| **Tissue** | | | **TCGA mutation not reported on COSMIC** | **Reference** |
| Pancreas | | | P325Pfs*48 | N/A |
| N/S | | | W126S, V325A, L173Wfs*17, F145Sfs*36, P435L, W270*, V352A, A442V, H179Y, K562N, Y358Sfs*14, R211H | N/A |

MAPS OF CDS MUTATIONS SHOWN IN THIS TABLE

Point and non-sense mutations


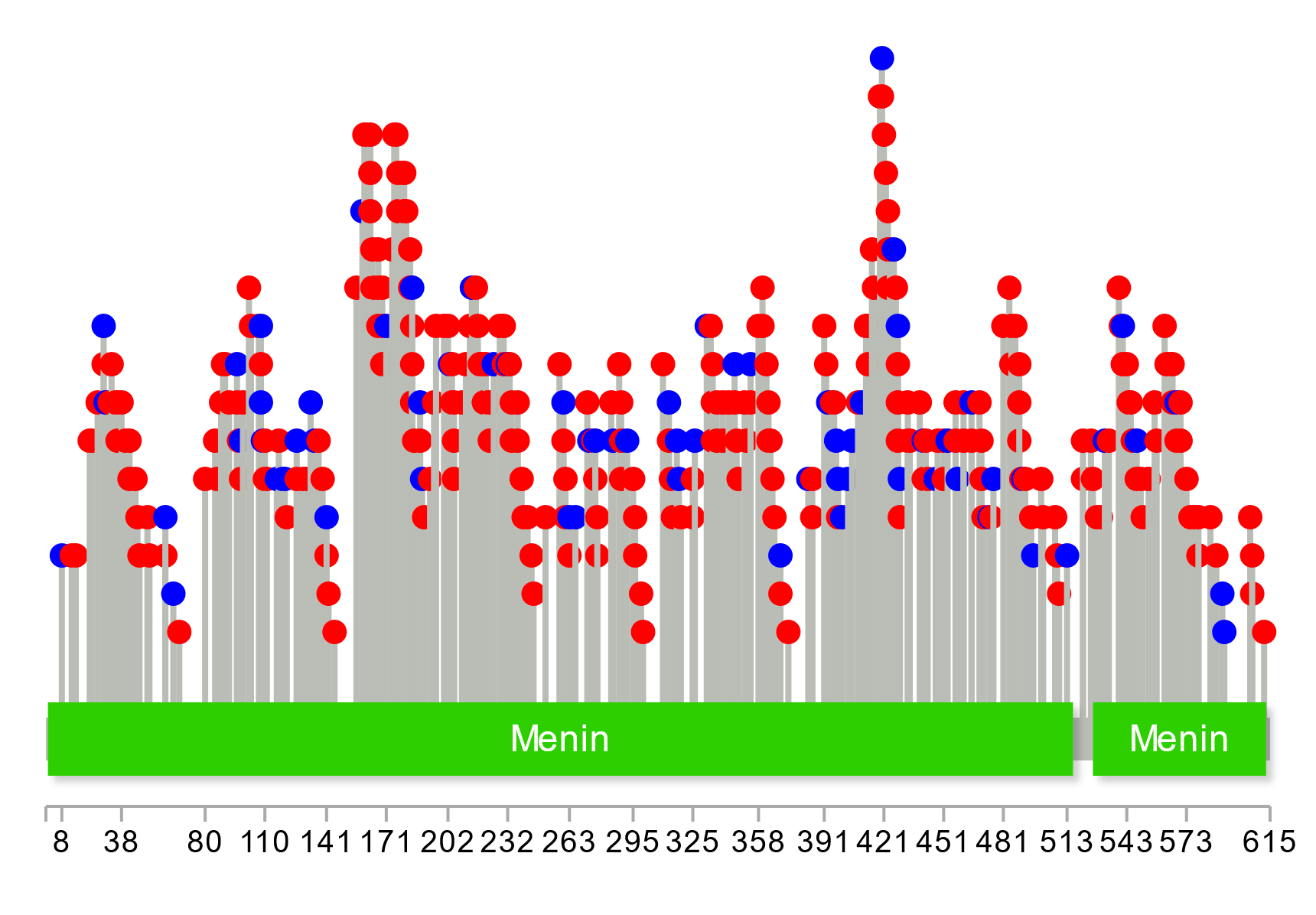


Frame-shift mutations


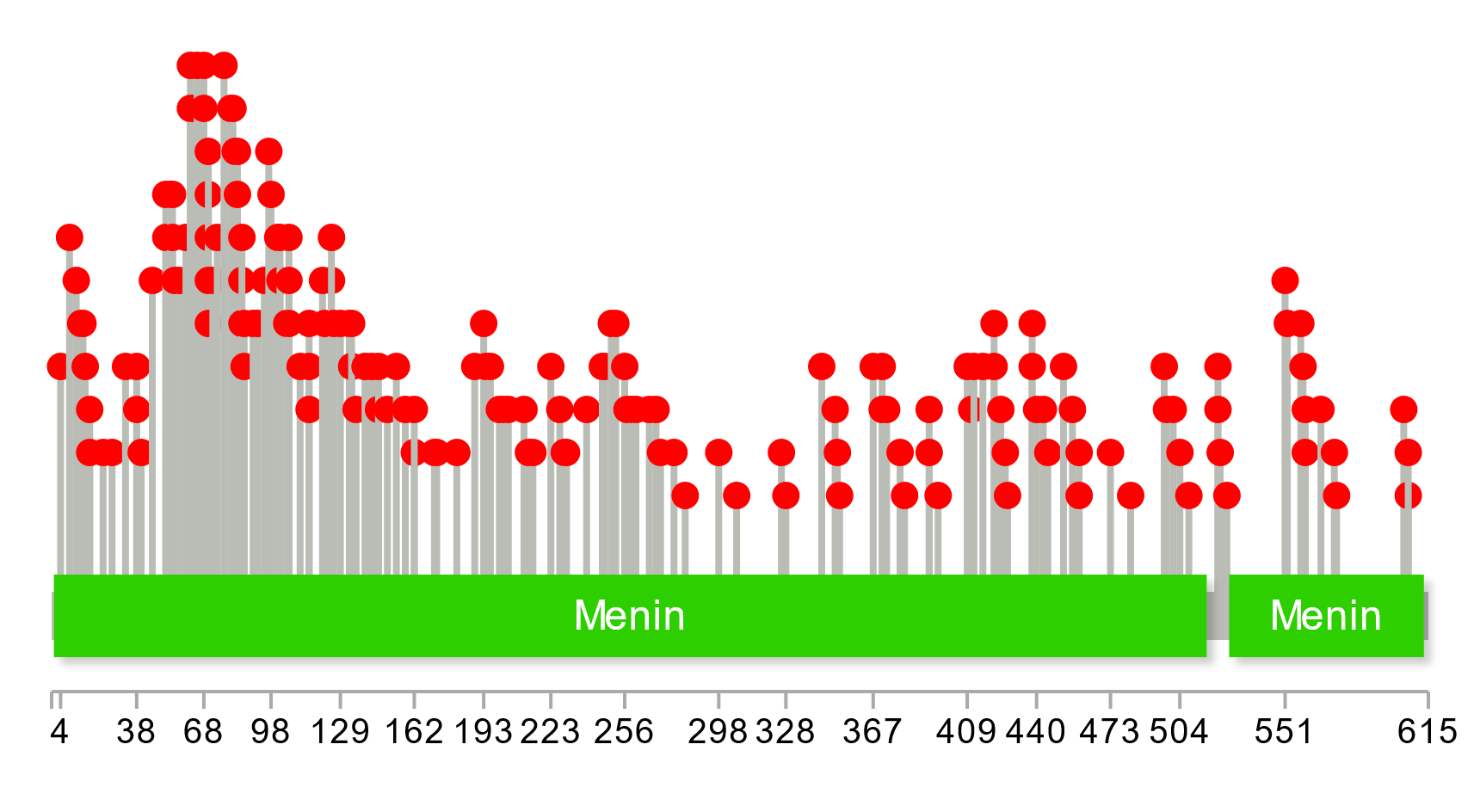


Deletion, insertion, complex deletion and insertion, and duplications


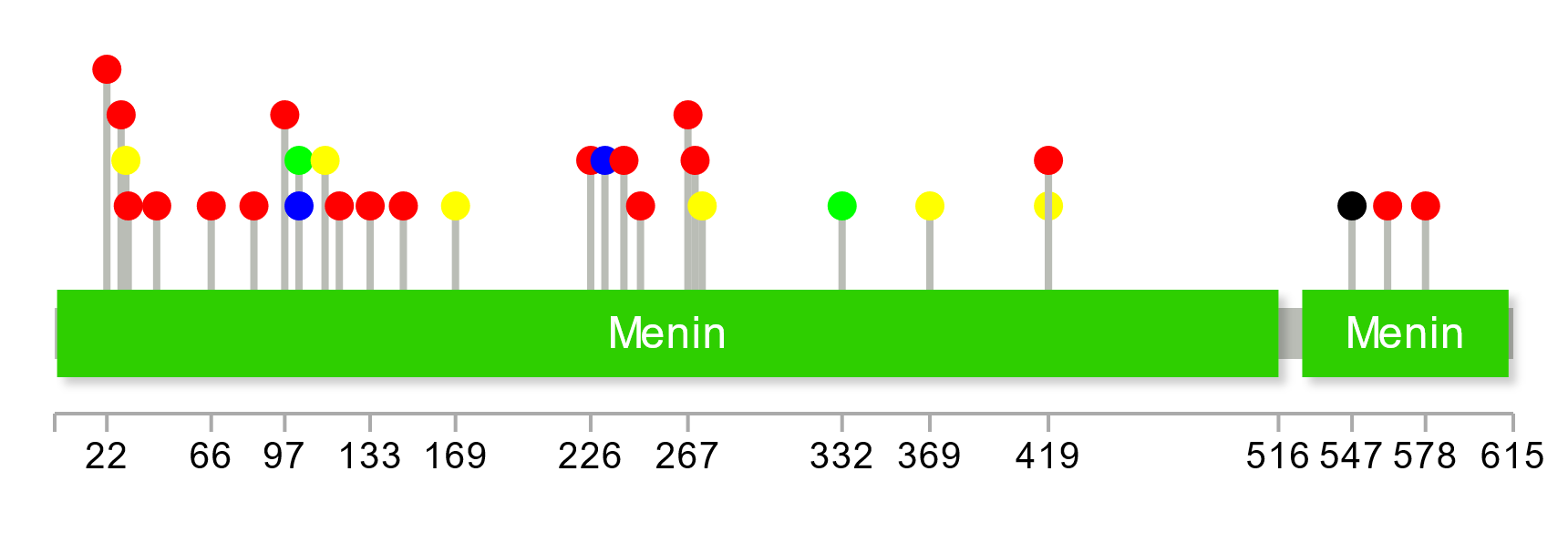


**Table S2.** ClinVar confirmed germline pathogenic mutations.

| **Mutation CDS** | **dbSNP Variant ID** |
| --- | --- |
| p.V50Cfs*67 | rs794728638 |
| p.T56Yfs*61 | rs794728656 |
| p.S313* | rs1565644366 |
| p.Y318* | rs1555165128 |
| p.D320Y | rs747851909 |
| p.H322fs | rs794728641 |
| p.D375fs | rs794728658 |
| p.Q403* | rs886039418 |
| p.Q405* | rs886039419 |
| p.Y422fs | rs1555164305 |
| p.E568* | rs794728632 |
| p.E568fs | rs398124436 |
| p.L556fs | rs794728645 |
| p.A540fs | rs794728660 |
| p.P545fs | rs794728660 |
| p.G536fs | rs886039421 |
| p.K522fs | rs761695866 |
| p.L509fs | rs1555163591 |
| p.K501fs | rs1565637724 |
| p.E482fs | rs886041214 |
| p.R465delinsT* | rs1064796889 |
| p.F452fs | rs794728643 |
| p.R451fs | rs886041746 |
| p.Q447* | rs794728654 |
| p.A165P | rs1565648656 |
| p.A242fs | rs1114167534 |
| p.A347fs | rs1555164986 |
| p.A373D | rs1555164707 |
| p.A469fs | rs1114167531, rs1114167500 |
| p.A515fs | rs1114167522 |
| p.A6fs | rs1114167523 |
| p.R108fs | rs1565651568 |
| p.R108Qfs*11 | rs878855191 |
| p.R108G | rs794728647 |
| p.R108* | rs794728647 |
| p.R234fs | rs1114167519 |
| p.R280fs | rs1555165360 |
| p.R29G | rs794728615 |
| p.R29* | rs794728615 |
| p.R340fs | rs1114167506 |
| p.R420* | rs1060499974 |
| p.R465* | rs104894267 |
| p.R521fs | rs767319284 |
| p.R532* | rs104894261 |
| p.R92fs | rs1114167516 |
| p.N217fs | rs878855196 |
| p.N57fs | rs1060499990 |
| p.D177Y | rs1114167494 |
| p.D411fs | rs1114167513 |
| p.D423Y | rs104894264 |
| p.D423H | rs104894264 |
| p.D423N | rs104894264 |
| p.D70fs | rs1555166609, rs1057517902 |
| p.D70Pfs*51 | rs730882136 |
| p.C359* | rs104894265 |
| p.C414fs | rs1114167524 |
| p.Q141* | rs886039553 |
| p.Q214fs | rs1114167538 |
| p.Q214* | rs1565647767 |
| p.Q263E | rs886039416 |
| p.Q263* | rs886039416 |
| p.Q265* | rs104894266 |
| p.Q266* | rs1057520733 |
| p.Q398E | rs1060499984 |
| p.Q398* | rs1060499984 |
| p.Q410* | rs864622615 |
| p.Q455* | rs1114167509 |
| p.Q559* | rs794728631 |
| p.Q96Pfs*25 | rs1555166494 |
| p.E116K | rs1060499992 |
| p.E116* | rs1060499992 |
| p.E260K | rs104894268 |
| p.E260Q | rs104894268 |
| p.E341fs | rs1114167483 |
| p.E368del | rs869025185 |
| p.E397fs | rs386134247 |
| p.E429K | rs1114167477 |
| p.E429* | rs1114167477 |
| p.E397K | rs772588551 |
| p.E474fs | rs1555163780 |
| p.E496fs | rs1555163646 |
| p.E482* | rs863224526 |
| p.E482Q | rs863224526 |
| p.E552fs | rs1114167496 |
| p.E561* | rs1114167501 |
| p.G161D | rs794728648 |
| p.G161V | rs794728648 |
| p.G508fs | rs1114167476 |
| p.G450fs | rs1565640081 |
| p.G477fs | rs1114167536 |
| p.G553fs | rs1555163136 |
| p.H139R | rs1114167515 |
| p.H139D | rs104894263 |
| p.H139fs | rs1114167540 |
| p.I252fs | rs1555165503 |
| p.I248fs | rs1114167532 |
| p.I85fs | rs386134253, rs587776841 |
| p.L103fs | rs794728639 |
| p.L129fs | rs1565651223 |
| p.L173P | rs386134256 |
| p.L182fs | rs1114167470, rs1114167503 |
| p.L228P | rs886039415 |
| p.L22R | rs104894256 |
| p.L329fs | rs1114167508 |
| p.L48fs | rs1555166681 |
| p.L75fs | rs1114167485 |
| p.L89fs | rs1114167490 |
| p.L120del | rs794728657 |
| p.L120* | rs878855192 |
| p.L135* | rs121913034 |
| p.L135Q | rs121913034 |
| p.L238* | rs1114167499 |
| p.L493fs | rs886039420 |
| p.L522* | rs794728630 |
| p.L564fs | rs1565635941 |
| p.M1L | rs386134250 |
| p.M1V | rs386134250 |
| p.F134fs | rs397515385 |
| p.F144fs | rs1114167481 |
| p.F144* | rs1114167511 |
| p.F421fs | rs1114167542 |
| p.P193fs | rs1555165756 |
| p.P250fs | rs1565646772 |
| p.P325L | rs1114167469 |
| p.P71fs | rs386134251 |
| p.S113fs | rs1114167478 |
| p.S114fs | rs886041213 |
| p.S404fs | rs1555164430 |
| p.S464fs | rs1555163883 |
| p.S517L | rs141679530 |
| p.S517* | rs141679530 |
| p.S517fs | rs794728659 |
| p.S560fs | rs1114167510 |
| p.T215fs | rs794728640 |
| p.T530fs | rs1114167514 |
| p.W126* | rs1555166365 |
| p.W188* | rs794728650 |
| p.W203* | rs104894257, rs104894258 |
| p.W225* | rs1565647197, rs886039414 |
| p.W225C | rs886039414 |
| p.W346* | rs1114167482 |
| p.W428* | rs1114167533 |
| p.W441C | rs398124435 |
| p.W441* | rs104894260 |
| p.W441R | rs104894259 |
| p.W476* | rs1060499991 |
| p.Y106fs | rs1555166466 |
| p.Y106* | rs1060499987 |
| p.Y232* | rs778921501 |
| p.Y281* | rs1060503789 |
| p.Y317* | rs386134260 |
| p.Y328fs | rs1114167541 |
| p.Y328* | rs750904332 |
| p.Y356* | rs767078097 |
| p.Y77* | rs1555166567 |
| p.Y90* | rs1114167527 |
| p.V189E | rs104894262 |
| p.V80fs | rs1114167486 |
| p.Q171* | rs1565648511 |
| p.A169fs | rs886041634 |
| p.P144fs | rs1064793613 |
| p.L117fs | rs1555166387 |
| p.V112fs | rs1555166435 |
| p.R98* | rs886039413 |
| p.L36fs | rs1555166711 |
| p.L27fs | rs794728637 |
| p.V20fs | rs794728636 |
| p.V19fs | rs794728655 |
| p.I125_W126ins* | rs1114167492 |
| p.S448_L449ins* | rs1114167520 |
| p.P412fs | rs1592637440 |
| p.*616R | rs1565635212 |
| p.C170* | rs1592651767 |
| p.Y227* | rs1555165597 |
| p.D423fs | rs1592637081 |
| p.Q344* | rs1592643178 |
| p.T256fs | rs1592648830 |
| p.G74fs | rs1592659414 |
| p.A472fs | rs1592633463 |
| p.R280K | rs1187634059 |
| p.V372fs | rs1592640213 |
| p.K309fs | rs1592646226 |
| p.W270* | rs1592647398 |
| p.S258fs | rs1592648765 |
| p.E26* | rs28931612 |
| p.F11fs | rs1592661082 |
| p.A247fs | rs1592649069 |
| p.A385fs | rs1592640181 |
| p.D411_P412ins* | rs1592637455 |
| p.D136fs | rs1592657785 |
| p.T198fs | rs1592650986 |
| p.G391fs | rs1592640081 |
| p.K562fs | rs1592630079 |
| p.T443fs | rs1555164184 |
| p.E479fs | rs1592633378 |
| p.V124fs | rs1555166368 |
| p.S15fs | rs1592660983 |
|  |  |
| **Splice site variants (detected in COSMIC)** | **dbSNP Variant ID** |
| c.1366-2_*132del | rs1565634591 |
| c.1365+1_1365+11del | rs764570645 |
| c.1365+1G>A | rs863223311 |
| c.1065-2A>G | rs1565642765 |
| c.1064+1G>C | rs1114167489 |
| c.928-1G>A | rs1057518572 |
| c.928-2A>G | rs1114167498 |
| c.927+2T>C | rs1555165256 |
| c.927+1G>C | rs398124437 |
| c.840-1_843delinsTACCTAGAGGTT | rs1592646831 |
| c.839+1G>T | rs1060499976 |
| c.799-1G>C | rs1555165377 |
| c.799-2A>G | rs1114167472 |
| 798+1G>C | rs794728652 |
| c.670-1G>C | rs1592649615 |
| c.549+106G>T | rs794728622 |
| c.549+106del | rs1057518903 (germline status unspecified) |
| c.566T>A | rs104894262 |
| c.461-1G>C | rs1064793672 |
| c.461-2A>G | rs886042035 |
|  |  |
| **Silent mutations** | **dbSNP Variant ID** |
| p.R465= | rs104894267 |
| p.Y232= | rs778921501 |
| p.Y281= | rs1060503789 |

**Table S3–S5.** please view at the excel file.

**References**

1. Assie, G., et al., *Integrated genomic characterization of adrenocortical carcinoma.* Nat Genet, 2014. **46**(6): p. 607-12.

2. Gortz, B., et al., *MEN1 gene mutation analysis of sporadic adrenocortical lesions.* Int J Cancer, 1999. **80**(3): p. 373-9.

3. Lippert, J., et al., *Targeted Molecular Analysis in Adrenocortical Carcinomas: A Strategy Toward Improved Personalized Prognostication.* J Clin Endocrinol Metab, 2018. **103**(12): p. 4511-4523.

4. Lee, H., et al., *Comprehensive genomic profiling of extrahepatic cholangiocarcinoma reveals a long tail of therapeutic targets.* J Clin Pathol, 2016. **69**(5): p. 403-8.

5. Schulte, K.M., et al., *Complete sequencing and messenger ribonucleic acid expression analysis of the MEN I gene in adrenal cancer.* J Clin Endocrinol Metab, 2000. **85**(1): p. 441-8.

6. Pillai, S., et al., *Silent genetic alterations identified by targeted next-generation sequencing in pheochromocytoma/paraganglioma: A clinicopathological correlations.* Exp Mol Pathol, 2017. **102**(1): p. 41-46.

7. Schulte, K.M., et al., *MEN I gene mutations in sporadic adrenal adenomas.* Hum Genet, 1999. **105**(6): p. 603-10.

8. Heppner, C., et al., *Somatic mutation of the MEN1 gene in parathyroid tumours.* Nat Genet, 1997. **16**(4): p. 375-8.

9. Li, M., et al., *Whole-exome and targeted gene sequencing of gallbladder carcinoma identifies recurrent mutations in the ErbB pathway.* Nat Genet, 2014. **46**(8): p. 872-6.

10. Mimaki, S., et al., *Hypermutation and unique mutational signatures of occupational cholangiocarcinoma in printing workers exposed to haloalkanes.* Carcinogenesis, 2016. **37**(8): p. 817-826.

11. Zehir, A., et al., *Mutational landscape of metastatic cancer revealed from prospective clinical sequencing of 10,000 patients.* Nat Med, 2017. **23**(6): p. 703-713.

12. Wardell, C.P., et al., *Genomic characterization of biliary tract cancers identifies driver genes and predisposing mutations.* J Hepatol, 2018. **68**(5): p. 959-969.

13. Crompton, B.D., et al., *The genomic landscape of pediatric Ewing sarcoma.* Cancer Discov, 2014. **4**(11): p. 1326-41.

14. Totoki, Y., et al., *Unique mutation portraits and frequent COL2A1 gene alteration in chondrosarcoma.* Genome Res, 2014. **24**(9): p. 1411-20.

15. Hyman, D.M., et al., *HER kinase inhibition in patients with HER2- and HER3-mutant cancers.* Nature, 2018. **554**(7691): p. 189-194.

16. Lefebvre, C., et al., *Mutational Profile of Metastatic Breast Cancers: A Retrospective Analysis.* PLoS Med, 2016. **13**(12): p. e1002201.

17. Li, Z., et al., *Loss of the FAT1 Tumor Suppressor Promotes Resistance to CDK4/6 Inhibitors via the Hippo Pathway.* Cancer Cell, 2018. **34**(6): p. 893-905 e8.

18. Ferrari, A., et al., *A whole-genome sequence and transcriptome perspective on HER2-positive breast cancers.* Nat Commun, 2016. **7**: p. 12222.

19. Yap, Y.S., et al., *Elucidating therapeutic molecular targets in premenopausal Asian women with recurrent breast cancers.* NPJ Breast Cancer, 2018. **4**: p. 19.

20. Nik-Zainal, S., et al., *Landscape of somatic mutations in 560 breast cancer whole-genome sequences.* Nature, 2016. **534**(7605): p. 47-54.

21. Fu, Y., et al., *Improving the Performance of Somatic Mutation Identification by Recovering Circulating Tumor DNA Mutations.* Cancer Res, 2016. **76**(20): p. 5954-5961.

22. Lee, J.K., et al., *Spatiotemporal genomic architecture informs precision oncology in glioblastoma.* Nat Genet, 2017. **49**(4): p. 594-599.

23. Wu, G., et al., *The genomic landscape of diffuse intrinsic pontine glioma and pediatric non-brainstem high-grade glioma.* Nat Genet, 2014. **46**(5): p. 444-450.

24. Gao, Y.B., et al., *Genetic landscape of esophageal squamous cell carcinoma.* Nat Genet, 2014. **46**(10): p. 1097-102.

25. Kawamura, J., et al., *Multiple endocrine neoplasia type 1 gene mutations in sporadic gastrinomas in Japan.* Oncol Rep, 2005. **14**(1): p. 47-52.

26. Goebel, S.U., et al., *Genotype/phenotype correlation of multiple endocrine neoplasia type 1 gene mutations in sporadic gastrinomas.* J Clin Endocrinol Metab, 2000. **85**(1): p. 116-23.

27. Mailman, M.D., et al., *Identification of MEN1 mutations in sporadic enteropancreatic neuroendocrine tumors by analysis of paraffin-embedded tissue.* Clin Chem, 1999. **45**(1): p. 29-34.

28. Toliat, M.R., et al., *Mutations in the MEN I gene in sporadic neuroendocrine tumours of gastroenteropancreatic system.* Lancet, 1997. **350**(9086): p. 1223.

29. Malouf, G.G., et al., *Genomic Characterization of Renal Cell Carcinoma with Sarcomatoid Dedifferentiation Pinpoints Recurrent Genomic Alterations.* Eur Urol, 2016. **70**(2): p. 348-57.

30. Giannakis, M., et al., *RNF43 is frequently mutated in colorectal and endometrial cancers.* Nat Genet, 2014. **46**(12): p. 1264-6.

31. Giannakis, M., et al., *Genomic Correlates of Immune-Cell Infiltrates in Colorectal Carcinoma.* Cell Rep, 2016. **15**(4): p. 857-865.

32. Cancer Genome Atlas, N., *Comprehensive molecular characterization of human colon and rectal cancer.* Nature, 2012. **487**(7407): p. 330-7.

33. Mouradov, D., et al., *Colorectal cancer cell lines are representative models of the main molecular subtypes of primary cancer.* Cancer Res, 2014. **74**(12): p. 3238-47.

34. Chakrabarty, S., et al., *Targeted sequencing-based analyses of candidate gene variants in ulcerative colitis-associated colorectal neoplasia.* Br J Cancer, 2017. **117**(1): p. 136-143.

35. van de Wetering, M., et al., *Prospective derivation of a living organoid biobank of colorectal cancer patients.* Cell, 2015. **161**(4): p. 933-45.

36. Liu, Z., et al., *The landscape of somatic mutation in sporadic Chinese colorectal cancer.* Oncotarget, 2018. **9**(44): p. 27412-27422.

37. Han, S.W., et al., *Targeted sequencing of cancer-related genes in colorectal cancer using next-generation sequencing.* PLoS One, 2013. **8**(5): p. e64271.

38. Swarts, D.R., et al., *MEN1 gene mutation and reduced expression are associated with poor prognosis in pulmonary carcinoids.* J Clin Endocrinol Metab, 2014. **99**(2): p. E374-8.

39. McMillan, E.A., et al., *Chemistry-First Approach for Nomination of Personalized Treatment in Lung Cancer.* Cell, 2018. **173**(4): p. 864-878 e29.

40. Veschi, S., et al., *Alterations of MEN1 and E-cadherin/beta-catenin complex in sporadic pulmonary carcinoids.* Int J Oncol, 2012. **41**(4): p. 1221-8.

41. Imielinski, M., et al., *Mapping the hallmarks of lung adenocarcinoma with massively parallel sequencing.* Cell, 2012. **150**(6): p. 1107-20.

42. Gortz, B., et al., *Mutations and allelic deletions of the MEN1 gene are associated with a subset of sporadic endocrine pancreatic and neuroendocrine tumors and not restricted to foregut neoplasms.* Am J Pathol, 1999. **154**(2): p. 429-36.

43. Fernandez-Cuesta, L., et al., *Frequent mutations in chromatin-remodelling genes in pulmonary carcinoids.* Nat Commun, 2014. **5**: p. 3518.

44. Abaan, O.D., et al., *The exomes of the NCI-60 panel: a genomic resource for cancer biology and systems pharmacology.* Cancer Res, 2013. **73**(14): p. 4372-82.

45. Seo, J.S., et al., *The transcriptional landscape and mutational profile of lung adenocarcinoma.* Genome Res, 2012. **22**(11): p. 2109-19.

46. Kan, Z., et al., *Diverse somatic mutation patterns and pathway alterations in human cancers.* Nature, 2010. **466**(7308): p. 869-73.

47. Shi, J., et al., *Somatic Genomics and Clinical Features of Lung Adenocarcinoma: A Retrospective Study.* PLoS Med, 2016. **13**(12): p. e1002162.

48. Debelenko, L.V., et al., *Identification of MEN1 gene mutations in sporadic carcinoid tumors of the lung.* Hum Mol Genet, 1997. **6**(13): p. 2285-90.

49. Debelenko, L.V., et al., *MEN1 gene mutation analysis of high-grade neuroendocrine lung carcinoma.* Genes Chromosomes Cancer, 2000. **28**(1): p. 58-65.

50. Ding, L., et al., *Somatic mutations affect key pathways in lung adenocarcinoma.* Nature, 2008. **455**(7216): p. 1069-75.

51. Uchino, S., et al., *Screening of the Men1 gene and discovery of germ-line and somatic mutations in apparently sporadic parathyroid tumors.* Cancer Res, 2000. **60**(19): p. 5553-7.

52. Cancer Genome Atlas Research, N., *Integrated genomic analyses of ovarian carcinoma.* Nature, 2011. **474**(7353): p. 609-15.

53. Mueller, J.J., et al., *Massively parallel sequencing analysis of mucinous ovarian carcinomas: genomic profiling and differential diagnoses.* Gynecol Oncol, 2018. **150**(1): p. 127-135.

54. Hoogstraat, M., et al., *Genomic and transcriptomic plasticity in treatment-naive ovarian cancer.* Genome Res, 2014. **24**(2): p. 200-11.

55. Jiao, Y., et al., *DAXX/ATRX, MEN1, and mTOR pathway genes are frequently altered in pancreatic neuroendocrine tumors.* Science, 2011. **331**(6021): p. 1199-203.

56. Chou, W.C., et al., *Genes involved in angiogenesis and mTOR pathways are frequently mutated in Asian patients with pancreatic neuroendocrine tumors.* Int J Biol Sci, 2016. **12**(12): p. 1523-1532.

57. Zhuang, Z., et al., *Somatic mutations of the MEN1 tumor suppressor gene in sporadic gastrinomas and insulinomas.* Cancer Res, 1997. **57**(21): p. 4682-6.

58. Wang, E.H., et al., *Mutation of the MENIN gene in sporadic pancreatic endocrine tumors.* Cancer Res, 1998. **58**(19): p. 4417-20.

59. Bergman, L., et al., *Identification of somatic mutations of the MEN1 gene in sporadic endocrine tumours.* Br J Cancer, 2000. **83**(8): p. 1003-8.

60. Backman, S., et al., *Detection of Somatic Mutations in Gastroenteropancreatic Neuroendocrine Tumors Using Targeted Deep Sequencing.* Anticancer Res, 2017. **37**(2): p. 705-712.

61. Cetani, F., et al., *Six novel MEN1 gene mutations in sporadic parathyroid tumors.* Hum Mutat, 2000. **16**(5): p. 445.

62. Jakel, C., et al., *Genome-wide genetic and epigenetic analyses of pancreatic acinar cell carcinomas reveal aberrations in genome stability.* Nat Commun, 2017. **8**(1): p. 1323.

63. Gonzalez-Vela, M.D.C., et al., *Shared Oncogenic Pathways Implicated in Both Virus-Positive and UV-Induced Merkel Cell Carcinomas.* J Invest Dermatol, 2017. **137**(1): p. 197-206.

64. Asteria, C., et al., *MEN1 gene mutations are a rare event in patients with sporadic neuroendocrine tumors.* Eur J Intern Med, 2002. **13**(5): p. 319-323.

65. Okauchi, Y., et al., *Glucagonoma diagnosed by arterial stimulation and venous sampling (ASVS).* Intern Med, 2009. **48**(12): p. 1025-30.

66. Kimura, N., et al., *Multiple endocrine neoplasia type 1-associated cystic pancreatic endocrine neoplasia and multifocal cholesterol granulomas.* Pathol Int, 2010. **60**(4): p. 321-5.

67. Fujii, T., et al., *MEN1 gene mutations in sporadic neuroendocrine tumors of foregut derivation.* Pathol Int, 1999. **49**(11): p. 968-73.

68. Cromer, M.K., et al., *Identification of somatic mutations in parathyroid tumors using whole-exome sequencing.* J Clin Endocrinol Metab, 2012. **97**(9): p. E1774-81.

69. Pardi, E., et al., *Aryl hydrocarbon receptor interacting protein (AIP) mutations occur rarely in sporadic parathyroid adenomas.* J Clin Endocrinol Metab, 2013. **98**(7): p. 2800-10.

70. Newey, P.J., et al., *Whole-exome sequencing studies of nonhereditary (sporadic) parathyroid adenomas.* J Clin Endocrinol Metab, 2012. **97**(10): p. E1995-2005.

71. Scarpelli, D., et al., *Novel somatic MEN1 gene alterations in sporadic primary hyperparathyroidism and correlation with clinical characteristics.* J Endocrinol Invest, 2004. **27**(11): p. 1015-21.

72. Dwight, T., et al., *Loss of heterozygosity in sporadic parathyroid tumours: involvement of chromosome 1 and the MEN1 gene locus in 11q13.* Clin Endocrinol (Oxf), 2000. **53**(1): p. 85-92.

73. Tanaka, C., et al., *Biallelic inactivation by somatic mutations of the MEN1 gene in sporadic parathyroid tumors.* Cancer Lett, 2002. **175**(2): p. 175-9.

74. Sulaiman, L., et al., *Genetic characterization of large parathyroid adenomas.* Endocr Relat Cancer, 2012. **19**(3): p. 389-407.

75. Juhlin, C., et al., *Loss of parafibromin expression in a subset of parathyroid adenomas.* Endocr Relat Cancer, 2006. **13**(2): p. 509-23.

76. Sato, K., et al., *Somatic mutations of the multiple endocrine neoplasia type 1 (MEN1) gene in patients with sporadic, nonfamilial primary hyperparathyroidism.* Surgery, 2000. **127**(3): p. 337-41.

77. Farnebo, F., et al., *Alterations of the MEN1 gene in sporadic parathyroid tumors.* J Clin Endocrinol Metab, 1998. **83**(8): p. 2627-30.

78. Forsberg, L., et al., *Homozygous inactivation of the MEN1 gene as a specific somatic event in a case of secondary hyperparathyroidism.* Eur J Endocrinol, 2001. **145**(4): p. 415-20.

79. Miedlich, S., et al., *Frequency of somatic MEN1 gene mutations in monoclonal parathyroid tumours of patients with primary hyperparathyroidism.* Eur J Endocrinol, 2000. **143**(1): p. 47-54.

80. Haven, C.J., et al., *Identification of MEN1 and HRPT2 somatic mutations in paraffin-embedded (sporadic) parathyroid carcinomas.* Clin Endocrinol (Oxf), 2007. **67**(3): p. 370-6.

81. Tahara, H., et al., *Rare somatic inactivation of the multiple endocrine neoplasia type 1 gene in secondary hyperparathyroidism of uremia.* J Clin Endocrinol Metab, 2000. **85**(11): p. 4113-7.

82. Alvelos, M.I., et al., *MEN1 intragenic deletions may represent the most prevalent somatic event in sporadic primary hyperparathyroidism.* Eur J Endocrinol, 2013. **168**(2): p. 119-28.

83. Carling, T., et al., *Parathyroid MEN1 gene mutations in relation to clinical characteristics of nonfamilial primary hyperparathyroidism.* J Clin Endocrinol Metab, 1998. **83**(8): p. 2960-3.

84. Sato, K., et al., *Somatic mutations of the MEN1 gene and microsatellite instability in a case of tertiary hyperparathyroidism occurring during high phosphate therapy for acquired, hypophosphatemic osteomalacia.* J Clin Endocrinol Metab, 2001. **86**(11): p. 5564-71.

85. Pannett, A.A. and R.V. Thakker, *Somatic mutations in MEN type 1 tumors, consistent with the Knudson "two-hit" hypothesis.* J Clin Endocrinol Metab, 2001. **86**(9): p. 4371-4.

86. Karges, W., et al., *Multiple endocrine neoplasia type 1 (MEN1) gene mutations in a subset of patients with sporadic and familial primary hyperparathyroidism target the coding sequence but spare the promoter region.* J Endocrinol, 2000. **166**(1): p. 1-9.

87. Enomoto, K., et al., *The surgical strategy and the molecular analysis of patients with parathyroid cancer.* World J Surg, 2010. **34**(11): p. 2604-10.

88. Zhuang, Z., et al., *Mutations of the MEN1 tumor suppressor gene in pituitary tumors.* Cancer Res, 1997. **57**(24): p. 5446-51.

89. Reincke, M., et al., *Mutations in the deubiquitinase gene USP8 cause Cushing's disease.* Nat Genet, 2015. **47**(1): p. 31-8.

90. Wenbin, C., et al., *Mutations of the MEN1 tumor suppressor gene in sporadic pituitary tumors.* Cancer Lett, 1999. **142**(1): p. 43-7.

91. Abida, W., et al., *Prospective Genomic Profiling of Prostate Cancer Across Disease States Reveals Germline and Somatic Alterations That May Affect Clinical Decision Making.* JCO Precis Oncol, 2017. **2017**.

92. Manson-Bahr, D., et al., *Mutation detection in formalin-fixed prostate cancer biopsies taken at the time of diagnosis using next-generation DNA sequencing.* J Clin Pathol, 2015. **68**(3): p. 212-7.

93. Kumar, A., et al., *Substantial interindividual and limited intraindividual genomic diversity among tumors from men with metastatic prostate cancer.* Nat Med, 2016. **22**(4): p. 369-78.

94. Taylor, B.S., et al., *Integrative genomic profiling of human prostate cancer.* Cancer Cell, 2010. **18**(1): p. 11-22.

95. Grasso, C.S., et al., *The mutational landscape of lethal castration-resistant prostate cancer.* Nature, 2012. **487**(7406): p. 239-43.

96. Sakurai, A., et al., *Unusual clinical and pathological presentation of a neuroendocrine tumor in a patient with multiple endocrine neoplasia type 1.* Endocr J, 2009. **56**(7): p. 887-95.

97. Wang, K., et al., *Profiling of 149 Salivary Duct Carcinomas, Carcinoma Ex Pleomorphic Adenomas, and Adenocarcinomas, Not Otherwise Specified Reveals Actionable Genomic Alterations.* Clin Cancer Res, 2016. **22**(24): p. 6061-6068.

98. Pickering, C.R., et al., *Mutational landscape of aggressive cutaneous squamous cell carcinoma.* Clin Cancer Res, 2014. **20**(24): p. 6582-92.

99. Krauthammer, M., et al., *Exome sequencing identifies recurrent somatic RAC1 mutations in melanoma.* Nat Genet, 2012. **44**(9): p. 1006-14.

100. Nord, B., et al., *Malignant melanoma in patients with multiple endocrine neoplasia type 1 and involvement of the MEN1 gene in sporadic melanoma.* Int J Cancer, 2000. **87**(4): p. 463-7.

101. Shain, A.H., et al., *Exome sequencing of desmoplastic melanoma identifies recurrent NFKBIE promoter mutations and diverse activating mutations in the MAPK pathway.* Nat Genet, 2015. **47**(10): p. 1194-9.

102. South, A.P., et al., *NOTCH1 mutations occur early during cutaneous squamous cell carcinogenesis.* J Invest Dermatol, 2014. **134**(10): p. 2630-2638.

103. Goebel, S.U., et al., *Identical clonality of sporadic gastrinomas at multiple sites.* Cancer Res, 2000. **60**(1): p. 60-3.

104. Yachida, S., et al., *Genomic Sequencing Identifies ELF3 as a Driver of Ampullary Carcinoma.* Cancer Cell, 2016. **29**(2): p. 229-40.

105. Banck, M.S., et al., *The genomic landscape of small intestine neuroendocrine tumors.* J Clin Invest, 2013. **123**(6): p. 2502-8.

106. Boni, R., et al., *Somatic mutations of the MEN1 tumor suppressor gene detected in sporadic angiofibromas.* J Invest Dermatol, 1998. **111**(3): p. 539-40.

107. Pantaleo, M.A., et al., *Genome-Wide Analysis Identifies MEN1 and MAX Mutations and a Neuroendocrine-Like Molecular Heterogeneity in Quadruple WT GIST.* Mol Cancer Res, 2017. **15**(5): p. 553-562.

108. Shankar, G.M., et al., *Sporadic hemangioblastomas are characterized by cryptic VHL inactivation.* Acta Neuropathol Commun, 2014. **2**: p. 167.

109. Dulak, A.M., et al., *Exome and whole-genome sequencing of esophageal adenocarcinoma identifies recurrent driver events and mutational complexity.* Nat Genet, 2013. **45**(5): p. 478-86.

110. Kim, T.M., et al., *The mutational burdens and evolutionary ages of early gastric cancers are comparable to those of advanced gastric cancers.* J Pathol, 2014. **234**(3): p. 365-74.

111. Kakiuchi, M., et al., *Recurrent gain-of-function mutations of RHOA in diffuse-type gastric carcinoma.* Nat Genet, 2014. **46**(6): p. 583-7.

112. Liu, J., et al., *Integrated exome and transcriptome sequencing reveals ZAK isoform usage in gastric cancer.* Nat Commun, 2014. **5**: p. 3830.

113. Wang, Y., et al., *Mutations of epigenetic regulatory genes are common in thymic carcinomas.* Sci Rep, 2014. **4**: p. 7336.

114. Pozdeyev, N., et al., *Genetic Analysis of 779 Advanced Differentiated and Anaplastic Thyroid Cancers.* Clin Cancer Res, 2018. **24**(13): p. 3059-3068.

115. Agrawal, N., et al., *Exomic sequencing of medullary thyroid cancer reveals dominant and mutually exclusive oncogenic mutations in RET and RAS.* J Clin Endocrinol Metab, 2013. **98**(2): p. E364-9.

116. Landa, I., et al., *Genomic and transcriptomic hallmarks of poorly differentiated and anaplastic thyroid cancers.* J Clin Invest, 2016. **126**(3): p. 1052-66.

117. Kasaian, K., et al., *MEN1 mutations in Hurthle cell (oncocytic) thyroid carcinoma.* J Clin Endocrinol Metab, 2015. **100**(4): p. E611-5.

118. Rathmann, S., et al., *Partial break in tolerance of NKG2A(-)/LIR-1(-) single KIR(+) NK cells early in the course of HLA-matched, KIR-mismatched hematopoietic cell transplantation.* Bone Marrow Transplant, 2017. **52**(8): p. 1144-1155.

119. India Project Team of the International Cancer Genome, C., *Mutational landscape of gingivo-buccal oral squamous cell carcinoma reveals new recurrently-mutated genes and molecular subgroups.* Nat Commun, 2013. **4**: p. 2873.

120. Li, Y.Y., et al., *Exome and genome sequencing of nasopharynx cancer identifies NF-kappaB pathway activating mutations.* Nat Commun, 2017. **8**: p. 14121.

121. Van Allen, E.M., et al., *The genetic landscape of clinical resistance to RAF inhibition in metastatic melanoma.* Cancer discovery, 2014. **4**(1): p. 94-109.

122. Ross, J.S., et al., *Nonamplification ERBB2 genomic alterations in 5605 cases of recurrent and metastatic breast cancer: An emerging opportunity for anti-HER2 targeted therapies.* Cancer, 2016. **122**(17): p. 2654-62.

123. Wang, H., et al., *Insights into beta cell regeneration for diabetes via integration of molecular landscapes in human insulinomas.* Nat Commun, 2017. **8**(1): p. 767.

124. Tanaka, C., et al., *Analysis of loss of heterozygosity on chromosome 11 and infrequent inactivation of the MEN1 gene in sporadic pituitary adenomas.* J Clin Endocrinol Metab, 1998. **83**(8): p. 2631-4.

125. Schmidt, M.C., et al., *Analysis of the MEN1 gene in sporadic pituitary adenomas.* J Pathol, 1999. **188**(2): p. 168-73.

126. Moelans, C.B., et al., *The molecular genetic make-up of male breast cancer.* Endocr Relat Cancer, 2019. **26**(10): p. 779-794.

127. Parsons, D.W., et al., *An integrated genomic analysis of human glioblastoma multiforme.* Science, 2008. **321**(5897): p. 1807-12.

128. Jones, D.T., et al., *Dissecting the genomic complexity underlying medulloblastoma.* Nature, 2012. **488**(7409): p. 100-5.

129. Zhang, L., et al., *Genomic analyses reveal mutational signatures and frequently altered genes in esophageal squamous cell carcinoma.* Am J Hum Genet, 2015. **96**(4): p. 597-611.

130. Cheng, C., et al., *Genomic analyses reveal FAM84B and the NOTCH pathway are associated with the progression of esophageal squamous cell carcinoma.* Gigascience, 2016. **5**: p. 1.

131. Chang, J., et al., *Genomic analysis of oesophageal squamous-cell carcinoma identifies alcohol drinking-related mutation signature and genomic alterations.* Nat Commun, 2017. **8**: p. 15290.

132. George, J., et al., *Comprehensive genomic profiles of small cell lung cancer.* Nature, 2015. **524**(7563): p. 47-53.

133. Peifer, M., et al., *Integrative genome analyses identify key somatic driver mutations of small-cell lung cancer.* Nat Genet, 2012. **44**(10): p. 1104-10.

134. Liu, J., et al., *Biological background of the genomic variations of cf-DNA in healthy individuals.* Ann Oncol, 2019. **30**(3): p. 464-470.

135. Dutton-Regester, K., et al., *Melanomas of unknown primary have a mutation profile consistent with cutaneous sun-exposed melanoma.* Pigment Cell Melanoma Res, 2013. **26**(6): p. 852-60.

| 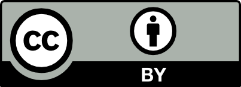 | © 2020 by the authors. Licensee MDPI, Basel, Switzerland. This article is an open access article distributed under the terms and conditions of the Creative Commons Attribution (CC BY) license (http://creativecommons.org/licenses/by/4.0/). |
| --- | --- |
